# Supplementary material for: Blood transcriptional correlates of BCG-induced protection against tuberculosis in rhesus macaques
Source: Cell Rep Med. 2023 Jun 29;4(7):101096. doi: 10.1016/j.xcrm.2023.101096 (PMC10394165; doi:10.1016/j.xcrm.2023.101096)
Supplement: Document S1. Tables S4–S6 and Figures S1–S5 [file mmc1.pdf]

Cell Reports Medicine, Volume *4*

Supplemental information

Blood transcriptional correlates of BCG-induced protection against tuberculosis in rhesus macaques

Yiran E. Liu, Patricia A. Darrah, Joseph J. Zeppa, Megha Kamath, Farida Laboune, Daniel C. Douek, Pauline Maiello, Mario Roederer, JoAnne L. Flynn, Robert A. Seder, and Purvesh Khatri

Table S4. Results of a non-linear model of module 1 scores on day 2 as a function of dose and protection outcome. To assess whether module 1 scores on day 2 were still associated with protection after adjusting for dose, we fit a non-linear least squares model using a generalized logistic function, akin to a dose-response curve with protection outcome as a covariate (see **STAR Methods:** Non-linear modeling adjusting for dose). The parameter β_A indicates how module 1 scores on day 2 differed by protection outcome, after accounting for dose.

Variable	Description	Estimate	P-value
A	lower asymptote	0.196	0.237
β_A	change if protected	0.663	<0.001
K	upper asymptote	1.441	<0.001
B	growth rate	2.731	0.142
M	dose midpoint	6.375	<0.001

Table S5. AUCs of all M1 genes or M1 sub-pathways in predicting protection following challenge. AUCs and confidence intervals were computed separately for high- and low-dose recipients. Related to Figure 3c-d.

Module or sub-pathway	AUC	CI_lower	CI_upper	Dose
Activated (LPS) dendritic cell surface signature (S11)	0.909	0.754	1.000	high
activated dendritic cells (M67)	0.841	0.626	1.000	high
All M1 genes	0.795	0.520	1.000	high
antiviral IFN signature (M75)	0.841	0.628	1.000	high
cell activation (IL15, IL23, TNF) (M24)	0.773	0.493	1.000	high
chemokines and inflammatory molecules in myeloid cells (M86.0)	0.659	0.221	1.000	high
DC surface signature (S5)	0.705	0.362	1.000	high
enriched in activated dendritic cells (II) (M165)	0.886	0.709	1.000	high
enriched in activated dendritic cells/monocytes (M64)	0.455	0.067	0.843	high
enriched in monocytes (II) (M11.0)	0.727	0.382	1.000	high
enriched in monocytes (IV) (M118.0)	0.591	0.187	0.995	high
immune activation - generic cluster (M37.0)	0.568	0.109	1.000	high
inflammasome receptors and signaling (M53)	0.682	0.355	1.000	high
innate activation by cytosolic DNA sensing (M13)	0.886	0.706	1.000	high
innate antiviral response (M150)	0.909	0.754	1.000	high
lysosome (M209)	0.682	0.298	1.000	high
myeloid, dendritic cell activation via NFkB (I) (M43.0)	0.795	0.509	1.000	high
myeloid, dendritic cell activation via NFkB (II) (M43.1)	0.364	0.000	0.798	high
proinflammatory cytokines and chemokines (M29)	0.727	0.416	1.000	high
Resting dendritic cell surface signature (S10)	0.636	0.189	1.000	high
RIG-1 like receptor signaling (M68)	0.955	0.852	1.000	high
type I interferon response (M127)	0.886	0.709	1.000	high
viral sensing & immunity; IRF2 targets network (I) (M111.0)	0.682	0.337	1.000	high
viral sensing & immunity; IRF2 targets network (II) (M111.1)	0.864	0.672	1.000	high
Activated (LPS) dendritic cell surface signature (S11)	0.958	0.866	1.000	low
activated dendritic cells (M67)	0.958	0.877	1.000	low
All M1 genes	0.986	0.948	1.000	low
antiviral IFN signature (M75)	0.972	0.908	1.000	low
cell activation (IL15, IL23, TNF) (M24)	0.944	0.826	1.000	low
chemokines and inflammatory molecules in myeloid cells (M86.0)	1.000	1.000	1.000	low
DC surface signature (S5)	0.917	0.769	1.000	low
enriched in activated dendritic cells (II) (M165)	0.972	0.907	1.000	low
enriched in activated dendritic cells/monocytes (M64)	0.944	0.844	1.000	low
enriched in monocytes (II) (M11.0)	0.861	0.586	1.000	low
enriched in monocytes (IV) (M118.0)	0.944	0.826	1.000	low
immune activation - generic cluster (M37.0)	1.000	1.000	1.000	low

inflammasome receptors and signaling (M53)	0.944	0.826	1.000	low
innate activation by cytosolic DNA sensing (M13)	0.958	0.877	1.000	low
innate antiviral response (M150)	0.944	0.830	1.000	low
lysosome (M209)	0.972	0.907	1.000	low
myeloid, dendritic cell activation via NFkB (I) (M43.0)	0.986	0.948	1.000	low
myeloid, dendritic cell activation via NFkB (II) (M43.1)	1.000	1.000	1.000	low
proinflammatory cytokines and chemokines (M29)	0.944	0.844	1.000	low
Resting dendritic cell surface signature (S10)	0.972	0.908	1.000	low
RIG-1 like receptor signaling (M68)	0.944	0.844	1.000	low
type I interferon response (M127)	0.903	0.762	1.000	low
viral sensing & immunity; IRF2 targets network (I) (M111.0)	1.000	1.000	1.000	low
viral sensing & immunity; IRF2 targets network (II) (M111.1)	0.944	0.843	1.000	low
Activated (LPS) dendritic cell surface signature (S11)	0.923	0.836	1.000	combined
activated dendritic cells (M67)	0.908	0.808	1.000	combined
All M1 genes	0.897	0.782	1.000	combined
antiviral IFN signature (M75)	0.908	0.806	1.000	combined
cell activation (IL15, IL23, TNF) (M24)	0.882	0.761	1.000	combined
chemokines and inflammatory molecules in myeloid cells (M86.0)	0.886	0.748	1.000	combined
DC surface signature (S5)	0.868	0.736	1.000	combined
enriched in activated dendritic cells (II) (M165)	0.923	0.835	1.000	combined
enriched in activated dendritic cells/monocytes (M64)	0.849	0.699	1.000	combined
enriched in monocytes (II) (M11.0)	0.853	0.708	0.998	combined
enriched in monocytes (IV) (M118.0)	0.857	0.709	1.000	combined
immune activation - generic cluster (M37.0)	0.871	0.720	1.000	combined
inflammasome receptors and signaling (M53)	0.875	0.745	1.000	combined
innate activation by cytosolic DNA sensing (M13)	0.908	0.808	1.000	combined
innate antiviral response (M150)	0.923	0.835	1.000	combined
lysosome (M209)	0.882	0.751	1.000	combined
myeloid, dendritic cell activation via NFkB (I) (M43.0)	0.897	0.781	1.000	combined
myeloid, dendritic cell activation via NFkB (II) (M43.1)	0.879	0.736	1.000	combined
proinflammatory cytokines and chemokines (M29)	0.882	0.760	1.000	combined
Resting dendritic cell surface signature (S10)	0.871	0.729	1.000	combined
RIG-1 like receptor signaling (M68)	0.923	0.837	1.000	combined
type I interferon response (M127)	0.904	0.806	1.000	combined
viral sensing & immunity; IRF2 targets network (I) (M111.0)	0.882	0.749	1.000	combined
viral sensing & immunity; IRF2 targets network (II) (M111.1)	0.904	0.803	1.000	combined

Table S6. Correlation between M1 sub-pathways and total Mtb CFU (log10) post-challenge. Pearson correlation coefficients and corresponding p-values are shown. Related to Figure 3e.

Module or sub-pathway	Cor.Coef	P.value
Activated (LPS) dendritic cell surface signature (S11)	-0.684	1.12E-05
activated dendritic cells (M67)	-0.680	1.36E-05
All M1 genes	-0.680	1.33E-05
antiviral IFN signature (M75)	-0.659	3.04E-05
cell activation (IL15, IL23, TNF) (M24)	-0.625	9.94E-05
chemokines and inflammatory molecules in myeloid cells (M86.0)	-0.647	4.67E-05
DC surface signature (S5)	-0.612	1.55E-04
enriched in activated dendritic cells (II) (M165)	-0.671	1.89E-05
enriched in activated dendritic cells/monocytes (M64)	-0.617	1.29E-04
enriched in monocytes (II) (M11.0)	-0.642	5.69E-05
enriched in monocytes (IV) (M118.0)	-0.615	1.41E-04
immune activation - generic cluster (M37.0)	-0.647	4.79E-05
inflammasome receptors and signaling (M53)	-0.668	2.16E-05
innate activation by cytosolic DNA sensing (M13)	-0.665	2.44E-05
innate antiviral response (M150)	-0.681	1.28E-05
lysosome (M209)	-0.688	9.65E-06
myeloid, dendritic cell activation via NFkB (I) (M43.0)	-0.621	1.16E-04
myeloid, dendritic cell activation via NFkB (II) (M43.1)	-0.608	1.75E-04
proinflammatory cytokines and chemokines (M29)	-0.604	1.95E-04
Resting dendritic cell surface signature (S10)	-0.604	1.97E-04
RIG-1 like receptor signaling (M68)	-0.697	6.49E-06
type I interferon response (M127)	-0.654	3.60E-05
viral sensing & immunity; IRF2 targets network (I) (M111.0)	-0.621	1.15E-04
viral sensing & immunity; IRF2 targets network (II) (M111.1)	-0.658	3.11E-05

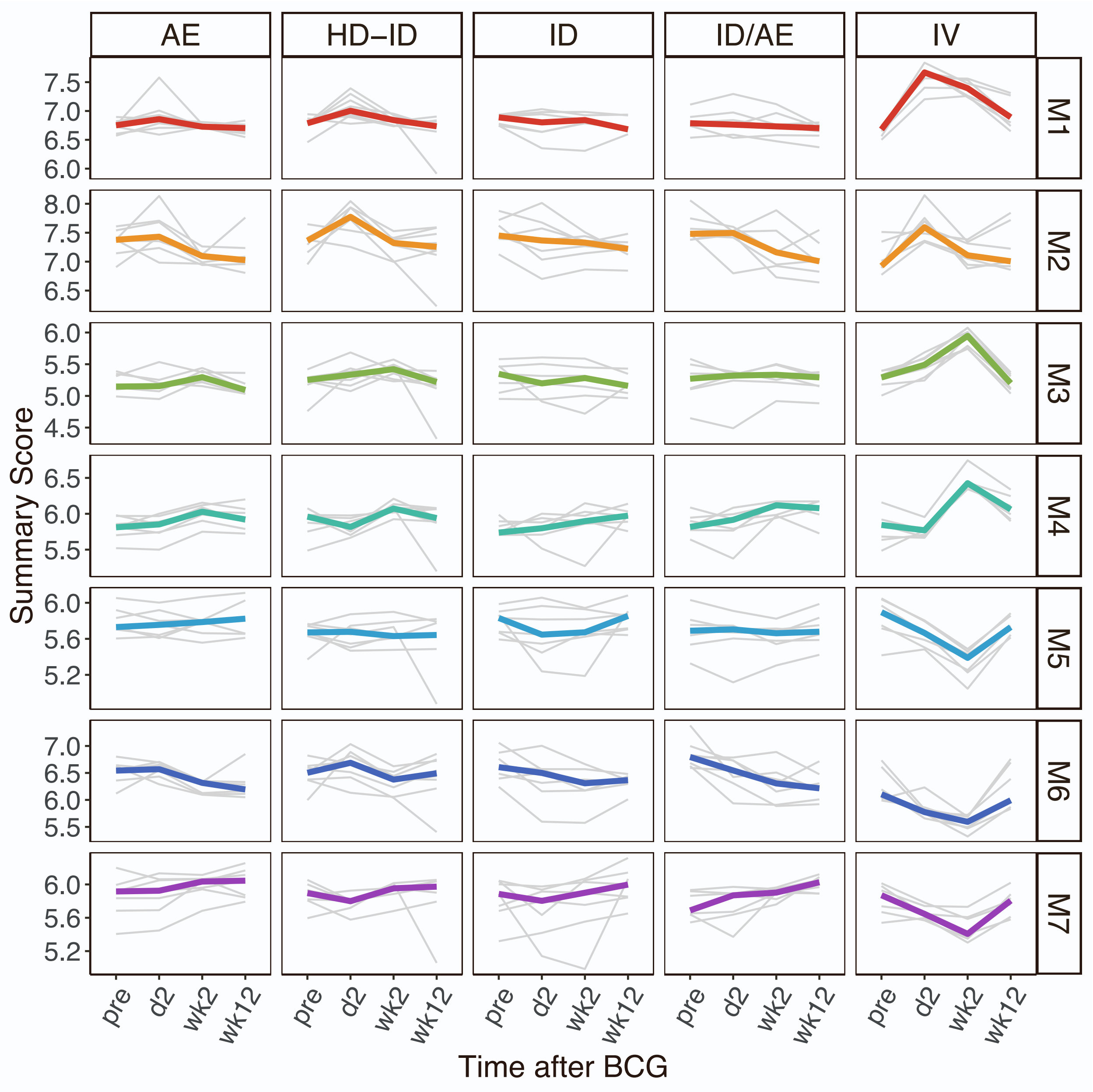

Figure S1. Module activity in macaques from the route study cohort. Summary scores of all seven modules over time following vaccination in the route study cohort, where macaques were BCG-vaccinated via different routes. AE, aerosol; HD-ID, high-dose intradermal; ID, intradermal; ID/AE, intradermal and aerosol; IV, high-dose intravenous. Shown are module scores for individual macaques (thin grey lines) and the median per group (thick colored lines). Related to Figure 1e-f.

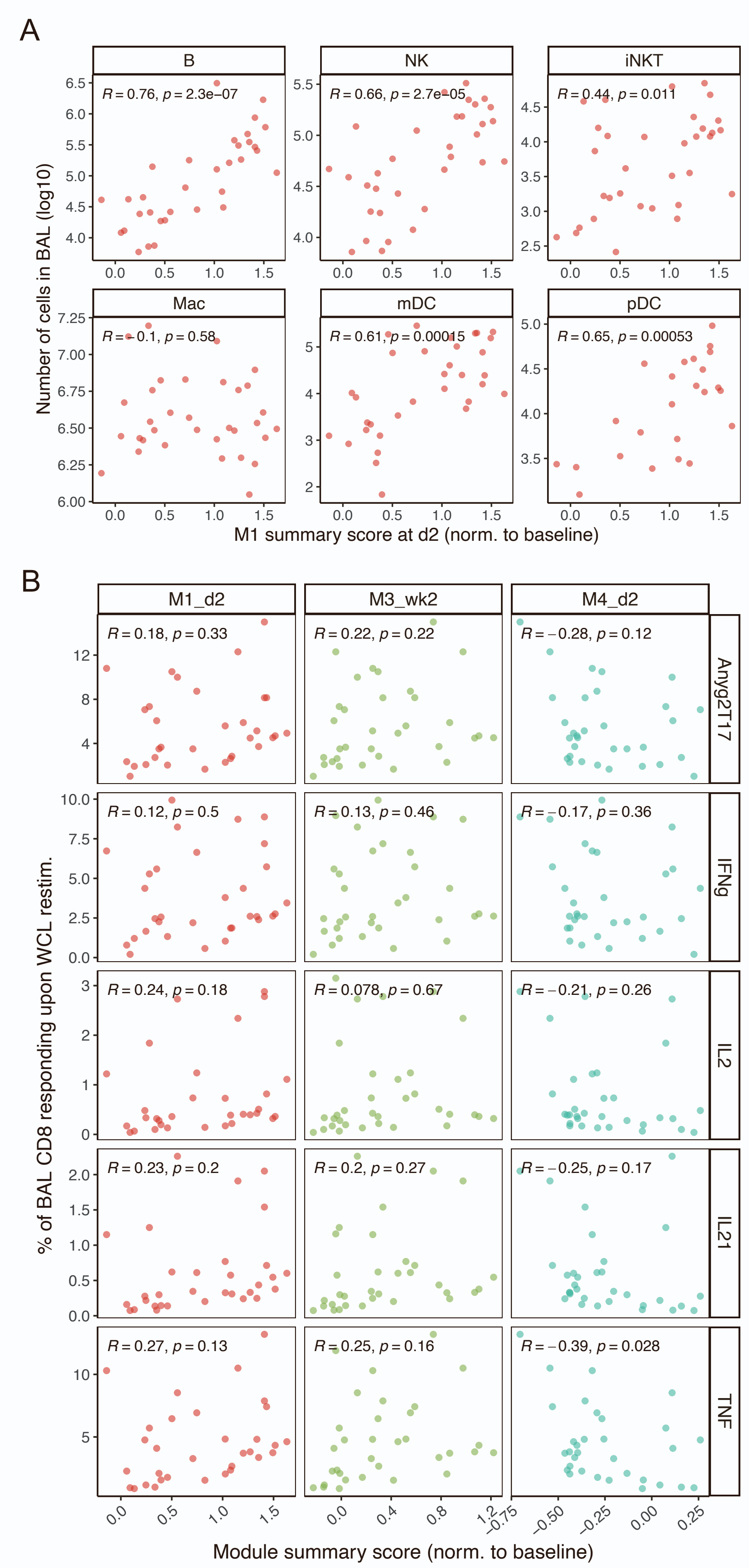
A
B
NK
iNKT
Mac
mDC
pDC
R = 0.76, p = 2.3e−07
R = 0.66, p = 2.7e−05
R = 0.44, p = 0.011
R = −0.1, p = 0.58
R = 0.61, p = 0.00015
R = 0.65, p = 0.00053
Number of cells in BAL (log10)
M1 summary score at d2 (norm. to baseline)
B
M1_d2
M3_wk2
M4_d2
Anyg2T17
IFNg
IL2
IL21
TNF
R = 0.18, p = 0.33
R = 0.22, p = 0.22
R = −0.28, p = 0.12
R = 0.12, p = 0.5
R = 0.13, p = 0.46
R = −0.17, p = 0.36
R = 0.24, p = 0.18
R = 0.078, p = 0.67
R = −0.21, p = 0.26
R = 0.23, p = 0.2
R = 0.2, p = 0.27
R = −0.25, p = 0.17
R = 0.27, p = 0.13
R = 0.25, p = 0.16
R = −0.39, p = 0.028
% of BAL CD8 responding upon WCL restim.
Module summary score (norm. to baseline)

Figure S2. Correlations between module scores in blood and immune responses in BAL. A) Correlations between module 1 scores at day 2 post-vaccination and number (log10) of B cells, NK cells, iNKT cells, macrophages, mDCs, or pDCs in BAL. All BAL cell counts were measured four weeks post-vaccination except NK cell counts which were measured eight weeks post-vaccination. pDC cell count measurements were missing for nine (26%) animals. B) Correlations between module 1 scores at day 2 post-vaccination, module 3 scores at week 2 post-vaccination, or module 4 scores at day 2 post-vaccination and the frequency of antigen-specific CD8 T cells in BAL at week 8 post-vaccination. Antigen-specific CD8 T cells are defined as the frequency of BAL CD8 T cells expressing a given cytokine or any combination of cytokines (Anyg2T17) upon *ex vivo* restimulation with *Mtb* whole cell lysate. Pearson correlation coefficients and corresponding p-values are shown. All module scores were normalized to each animal's baseline. Related to Figure 2.

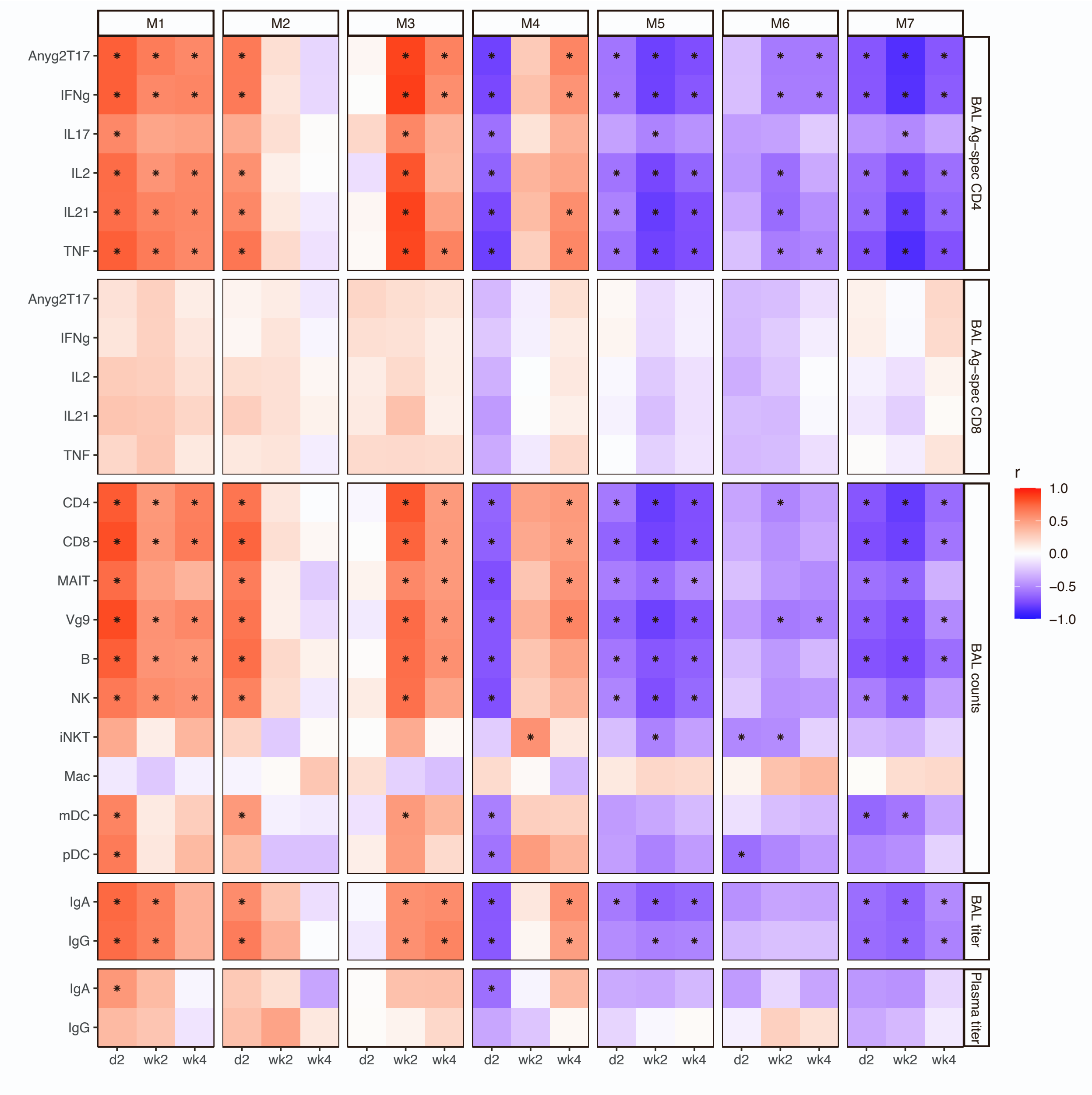

Figure S3. Complete correlation matrix for module scores in blood and immune responses in BAL or plasma. Pairwise pearson correlation coefficients (*r*) are shown between summary scores for each module at each timepoint and various immune responses of interest. Correlation p-values were adjusted using the Benjamini-Hochberg method; significant correlations at a false discovery rate threshold of 0.01 are indicated with an asterisk. BAL, bronchoalveolar lavage; Ag-spec, antigen-specific; Anyg2T17, expressing any combination of IFNγ, IL-2, TNF, IL-17. Antigen-specific CD4 and CD8 T cell responses are defined as the frequency of BAL CD4 or CD8 T cells expressing a given cytokine upon *ex vivo* restimulation with *Mtb* whole cell lysate eight weeks post-vaccination. BAL counts of various cell types and antibody titers are on a log10 scale and were measured four weeks post-vaccination, with the exception of NK cell counts which were measured eight weeks post-vaccination. pDC cell count measurements were missing for nine (26%) animals. All module scores were normalized to each animal's baseline. Related to Figure 2.

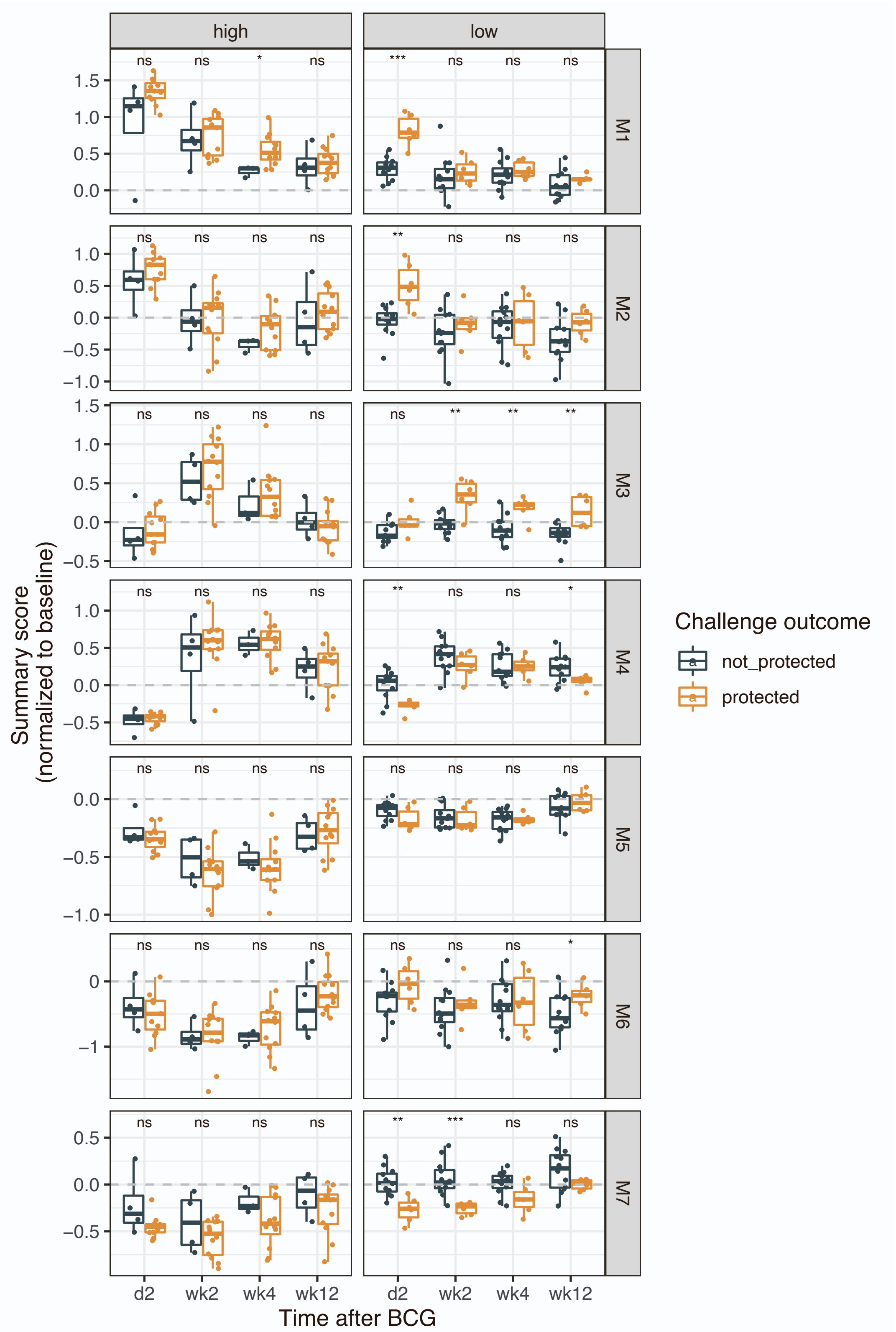

Figure S4. Module activity stratified by IV BCG dose and outcome following challenge. Summary scores (normalized to baseline) of all seven modules over time following vaccination, stratified by IV BCG dose and whether macaques were protected or not protected following *Mtb* challenge. The dotted horizontal line at y=0 indicates no change from baseline. Wilcoxon p-values are shown: *p ≤ 0.05, **p ≤ 0.01, ***p ≤ 0.001. Related to Figure 3b.

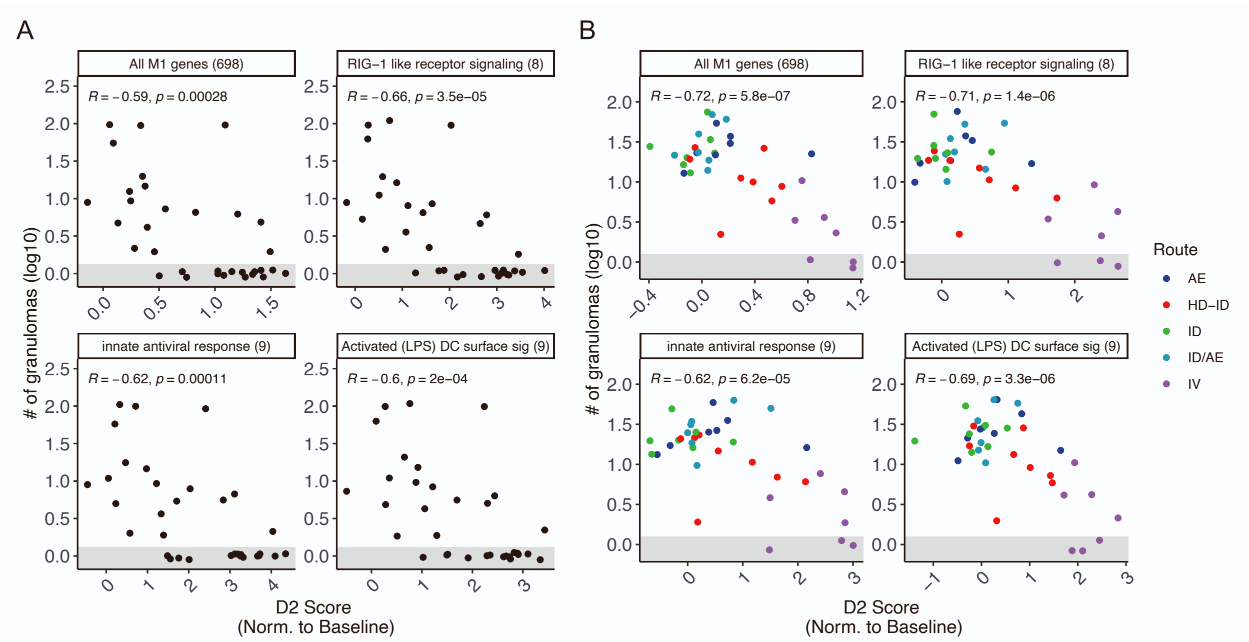

Figure S5. Correlations between the granuloma burden following challenge and module 1 pathways at day 2 post-vaccination. Correlations between the number of granulomas (log10) upon necropsy and day 2 scores of module 1 or its sub-pathways in the A) dose study cohort or B) route study cohort. Pearson correlation coefficients and corresponding p-values are shown. The number of genes in each set is shown in parentheses to the right of the set name. All scores were normalized to each animal's baseline. Data points are jittered to reduce overplotting; all points in the shaded grey area represent animals with no detectable CFUs. Related to Figure 3e-f.
