# Supplementary material for: Blood transcriptional correlates of BCG-induced protection against tuberculosis in rhesus macaques
Source: Cell Rep Med. 2023 Jun 29;4(7):101096. doi: 10.1016/j.xcrm.2023.101096 (PMC10394165; doi:10.1016/j.xcrm.2023.101096)
Supplement: Document S2. Article plus supplemental information [file mmc5.pdf]

Cell Reports
Medicine

Article

Blood transcriptional correlates of BCG-induced protection against tuberculosis in rhesus macaques

Graphical abstract

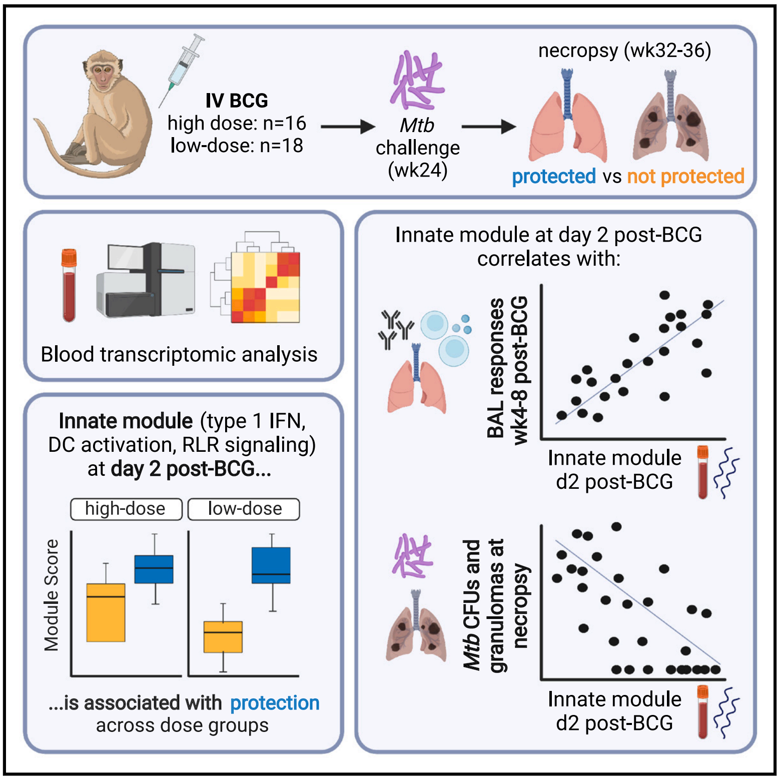

Authors

Yiran E. Liu, Patricia A. Darrah, Joseph J. Zeppa, ..., JoAnne L. Flynn, Robert A. Seder, Purvesh Khatri

Correspondence

pkhatri@stanford.edu

In brief

Liu et al. examine blood transcriptional responses to intravenous BCG vaccination in rhesus macaques and identify innate immune signatures induced 2 days after vaccination. These signatures correlate with antigen-specific immune responses in the lung 1–2 months later and predict protection against *Mtb* challenge 6 months later.

Highlights

- i.v. BCG vaccination induces innate and adaptive responses in the blood transcriptome
- An innate module at day 2 correlates with lung responses 1–2 months later
- Parsimonious signatures at day 2 predict protection against *Mtb* challenge

Liu et al., 2023, Cell Reports Medicine *4*, 101096
July 18, 2023 © 2023 The Author(s).
https://doi.org/10.1016/j.xcrm.2023.101096

CellPress

Cell Reports Medicine

CellPress
OPEN ACCESS

Article

Blood transcriptional correlates of BCG-induced protection against tuberculosis in rhesus macaques

Yiran E. Liu,[1,2] Patricia A. Darrah,[3] Joseph J. Zeppa,[4] Megha Kamath,[3] Farida Laboune,[3] Daniel C. Douek,[3] Pauline Maiello,[4] Mario Roederer,[3] JoAnne L. Flynn,[4] Robert A. Seder,[3] and Purvesh Khatri[1,5,6,7,*]
[1]Institute for Immunity, Transplantation and Infection, School of Medicine, Stanford University, Stanford, CA 94305, USA
[2]PhD Program in Epidemiology and Clinical Research, Department of Epidemiology and Population Health, Stanford University School of Medicine, Stanford, CA 94305, USA
[3]Vaccine Research Center, National Institute of Allergy and Infectious Diseases, National Institutes of Health, Bethesda, MD 20892, USA
[4]Department of Microbiology and Molecular Genetics and Center for Vaccine Research, University of Pittsburgh School of Medicine, Pittsburgh, PA 15213, USA
[5]Department of Biomedical Data Sciences, Stanford University, Stanford, CA 94305, USA
[6]Center for Biomedical Informatics Research, Department of Medicine, Stanford University, Stanford, CA 94305, USA
[7]Lead contact
*Correspondence: pkhatri@stanford.edu
https://doi.org/10.1016/j.xcrm.2023.101096

SUMMARY

Blood-based correlates of vaccine-induced protection against tuberculosis (TB) are urgently needed. Here, we analyze the blood transcriptome of rhesus macaques immunized with varying doses of intravenous (i.v.) BCG followed by *Mycobacterium tuberculosis* (*Mtb*) challenge. We use high-dose i.v. BCG recipients for "discovery" and validate our findings in low-dose recipients and in an independent cohort of macaques receiving BCG via different routes. We identify seven vaccine-induced gene modules, including an innate module (module 1) enriched for type 1 interferon and RIG-I-like receptor signaling pathways. Module 1 on day 2 post-vaccination highly correlates with lung antigen-responsive CD4 T cells at week 8 and with *Mtb* and granuloma burden following challenge. Parsimonious signatures within module 1 at day 2 post-vaccination predict protection following challenge with area under the receiver operating characteristic curve (AUROC) $\geq$0.91. Together, these results indicate that the early innate transcriptional response to i.v. BCG in peripheral blood may provide a robust correlate of protection against TB.

INTRODUCTION

Tuberculosis (TB) persists as a major contributor to global morbidity and mortality, with approximately 10 million new cases and 1.5 million deaths each year.[1] The only available TB vaccine, BCG, is widely administered at birth by the intradermal (ID) route but confers variable protection against adolescent and adult pulmonary TB.[2–4] An understanding of the vaccine-induced immune correlates of protection will guide future TB vaccine development.

Prior studies have focused on adaptive immune responses, especially antigen-specific polyfunctional CD4 T cells,[5,6] which have been shown in many animal models to be critical for vaccine-elicited protective immunity against *Mycobacterium tuberculosis* (*Mtb*). However, in humans vaccinated with BCG or the previously unsuccessful vaccine candidate MVA85A, CD4 T cell responses have failed to correlate with protection.[7–10] In one study, CD4 T cell responses were associated with TB disease risk but showed insufficient predictive accuracy; in fact, none of the 22 pre-specified immune variables evaluated in this study achieved areas under the receiver operating characteristic curves (AUROCs) over 0.66.[11] Thus, it is important to develop models of vaccination that confer high-level protection and to use analytical methods that more fully encompass the range of innate and adaptive responses to define more accurate correlates of protection.

Several new models of TB vaccination and challenge have achieved high levels of protection, which may enable more comprehensive approaches to identifying correlates of protection.[12–14] Notably, a recent study showed that intravenous delivery of BCG (i.v. BCG) conferred sterilizing protection against *Mtb* challenge in highly susceptible rhesus macaques.[12] Compared with ID or aerosol (AE) administration of BCG at the same dose, i.v. BCG elicited higher frequencies of systemic and lung-localized antigen-responsive CD4 and CD8 T cells, as well as increased counts of natural killer (NK) cells, mucosal-associated invariant T (MAIT) cells, and Vγ9+ $\gamma\delta$ T cells. In addition, i.v. BCG induced high antigen-specific antibody titers, including immunoglobulin M (IgM), in plasma and bronchoalveolar lavage (BAL).[15] While these findings suggest potential mechanisms by which i.v. BCG vaccination mediates protection compared with other routes, the high level of protection among i.v. BCG-vaccinated animals precluded identification of correlates of protection within this group. Moreover, in this study, there was no assessment of blood transcriptional responses to vaccination, which we and others have shown to be important

© 2023 The Author(s).
This is an open access article under the CC BY license (http://creativecommons.org/licenses/by/4.0/).

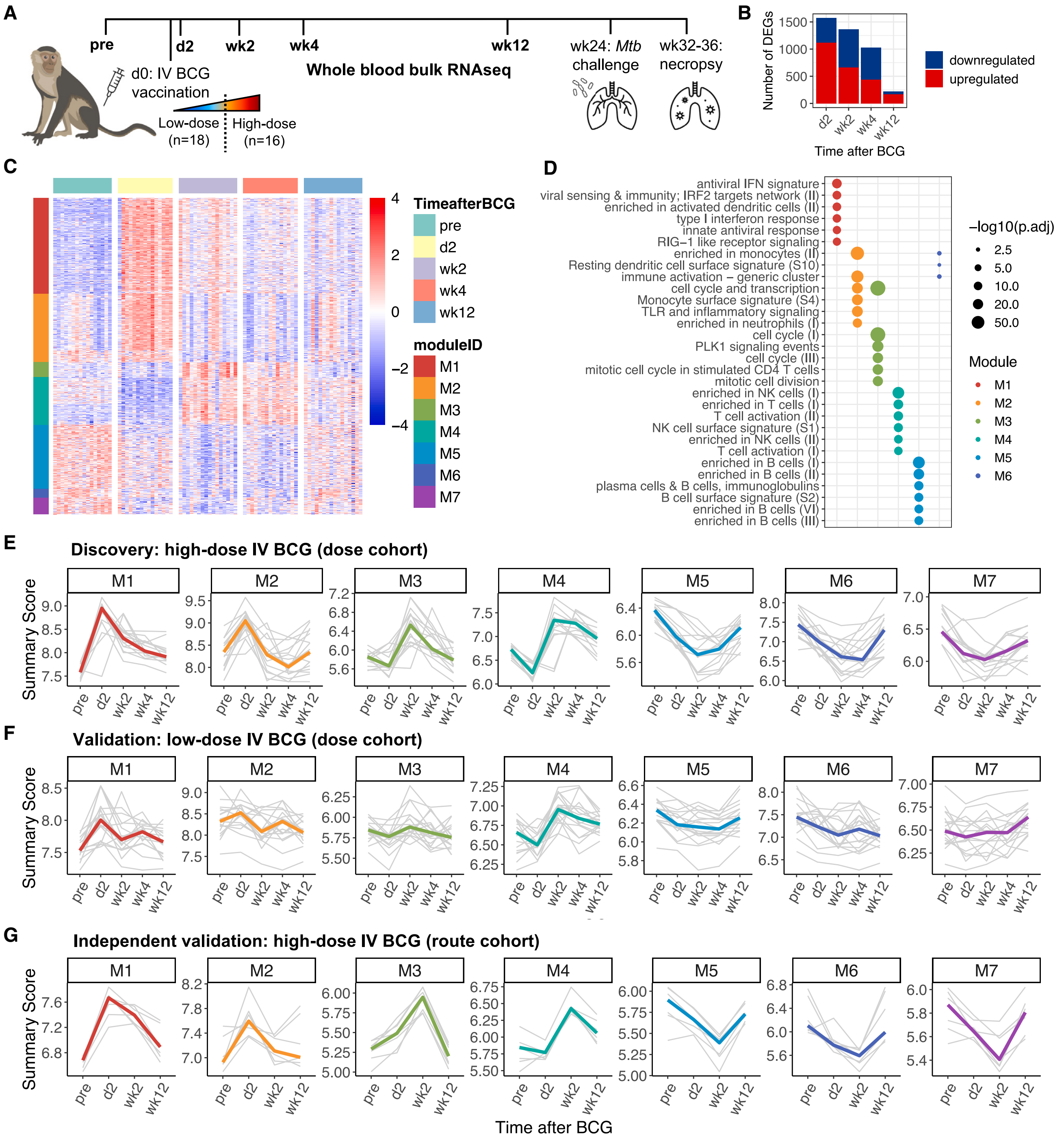

Figure 1. Blood transcriptional response to high-dose i.v. BCG vaccination

(A) Schematic of dose study. Whole-blood samples were collected at bolded time points for RNA sequencing. Macaques were classified as high-dose i.v. BCG recipients (n = 16) or low-dose i.v. BCG recipients (n = 18).

(B) Number of upregulated (red) or downregulated (blue) differentially expressed genes (DEGs) in high-dose i.v. BCG recipients at each time point relative to baseline.

(C) Heatmap of DEGs in high-dose recipients. Expression is scaled per row. Genes are organized by module. Unassigned genes (n = 191) are not shown.

(D) Significantly enriched (adjusted p value $\leq$ 0.01) immune pathways within each module. A maximum of six enriched pathways (ranked by significance) are shown for each module.

(legend continued on next page)

for identifying biomarkers of vaccine immunogenicity or efficacy against other infections including influenza, Ebola, and yellow fever.[16–19] Such transcriptomic analysis by our group has also provided a blood-based correlate of risk for TB in humans that continues to be independently validated and is actively progressing toward clinical translation.[20–22]

In the present study, we profiled the blood transcriptome in a new cohort of rhesus macaques that were vaccinated with varying doses of i.v. BCG to achieve a wider range of immune responses and outcomes following *Mtb* challenge. This "dose" cohort is detailed in a separate manuscript.[23] Here, we applied a systems vaccinology approach to comprehensively assess the blood transcriptional response to i.v. BCG across several time points following vaccination. We hypothesized that the blood transcriptional response would vary by dose but that distinct blood transcriptional signatures elicited by i.v. BCG vaccination would nonetheless predict localized immune responses in the lung and correlate with protection following *Mtb* challenge across dose groups. We then sought to validate these signatures in an independent cohort of macaques from the previously published study ("route" cohort)[12] to assess the generalizability of our signatures to other routes of BCG vaccination.

RESULTS

Thirty-four rhesus macaques of Indian origin were vaccinated with half-log increasing doses of i.v. BCG (between 3.9 × 10e4 and 2.5 × 10e7 colony-forming units [CFUs]) and challenged with *Mtb* 24 weeks later (Figure 1A). Bulk RNA sequencing was performed on whole-blood samples collected at baseline (pre-vaccination) and at 2 days, 2 weeks, 4 weeks, and 12 weeks following i.v. BCG vaccination (Figure 1A). All time points were prior to *Mtb* challenge. We were underpowered to perform high-dimensional multivariate analyses adjusting for time, vaccine dose, and protection outcome. However, we hypothesized that vaccine-induced and protection-associated responses would be most evident with high-dose i.v. BCG and could then be refined with low-dose i.v. BCG. Therefore, we analyzed high-dose ($>10^6$ CFU BCG; n = 16) and low-dose ($<10^6$ CFU BCG; n = 18) recipients separately as "discovery" and "validation" subsets, respectively (method details). Finally, we used blood transcriptional data from macaques in the route cohort as further independent validation.[12]

In the discovery subset of macaques that received high-dose i.v. BCG, 2,499 genes were differentially expressed (log2 fold change $\geq$ 1.5, false discovery rate [FDR] $\leq$ 10%) at one or more time points relative to baseline (Figures 1B and 1C). We note that we did not consider outcomes following *Mtb* challenge when identifying these differentially expressed genes (DEGs) in order to first analyze the overall response to i.v. BCG vaccination. Weighted gene correlation network analysis (WGCNA) of these 2,499 DEGs identified seven gene modules (Figure 1C; Table S1). We characterized each module using pathway analysis, with a reference gene set database specifically derived from studying blood transcriptional responses.[24] Modules 1 and 2 were upregulated early (day 2 post-vaccination) and enriched for dozens of innate immune pathways including those related to type 1 interferon (IFN) signaling, Toll-like receptor (TLR) and RIG-I-like receptor (RLR) signaling, dendritic cell (DC) and monocyte activation, and neutrophil recruitment (Figure 1D; Table S2). Modules 3 and 4 were upregulated later (2–4 weeks post-vaccination) and were enriched for pathways involved in CD4 T cell proliferation and T and NK cell activation. Finally, modules 5, 6, and 7 were downregulated following vaccination, with module 5 enriched for several B cell pathways and module 6 for a handful of DC and monocyte pathways. No pathways were significantly overrepresented in module 7, even when using a different reference gene set database, suggesting that it may be comprised of genes with unknown roles in the immune response to vaccination.

We summarized the activity of the seven modules over time using a score defined as the geometric mean expression of all genes in each module, and we modeled changes in module scores over time using generalized estimating equations (GEEs) (Figure 1E; Table S3; method details). GEE modeling showed that in the validation subset, low-dose i.v. BCG recipients exhibited similar trends (see statistical modeling of module expression over time in the STAR Methods details for details) in module activity over time as the discovery subset of high-dose i.v. BCG recipients for all modules except module 7 (Figure 1F; Table S3). However, the magnitude of vaccination-induced changes in module scores in low-dose recipients was lower and more variable. In extended validation, GEE results indicated that high-dose i.v.-vaccinated macaques from the independent route cohort exhibited similar trends in module scores over time for all modules but module 3 (Figure 1G; Table S3). For animals in the previous study that received BCG via other routes, changes in module scores over time were considerably dampened or even undetectable (Figure S1).

McCaffrey et al. recently demonstrated high concordance between localized immune responses in lung and systemic immune responses to *Mtb* infection in peripheral blood.[25] Further, in studies of vaccines against other bacterial and viral infections, blood transcriptional signatures measured just 1–3 days following vaccination correlated with subsequent antibody responses.[18,24] Therefore, we investigated whether blood transcriptional responses to i.v. BCG were correlated with immunological markers in BAL samples collected 4–8 weeks following vaccination (Figure 2A). These BAL features were examined by flow cytometry or Luminex independently of all blood transcriptomic analyses (Darrah et al., in press). Specifically, we hypothesized that early transcriptional responses to i.v. BCG in peripheral blood would be associated with subsequent adaptive responses, including those in lung.

Indeed, innate module 1 scores in blood on day 2 post-vaccination were highly correlated with CD4, CD8, MAIT, and γ9 T cell numbers, identified by flow cytometry in BAL 4 weeks post-vaccination (Pearson correlation coefficient $r \geq .72$,

(E–G) Summary scores representing the activity of each of the seven modules for individual macaques (thin gray lines) and median responses (thick colored lines) among (E) high-dose recipients from the dose cohort, (F) low-dose recipients from the dose cohort, and (G) high-dose recipients from the independent route cohort (n = 7). Animals from the route cohort were not sampled at week 4.

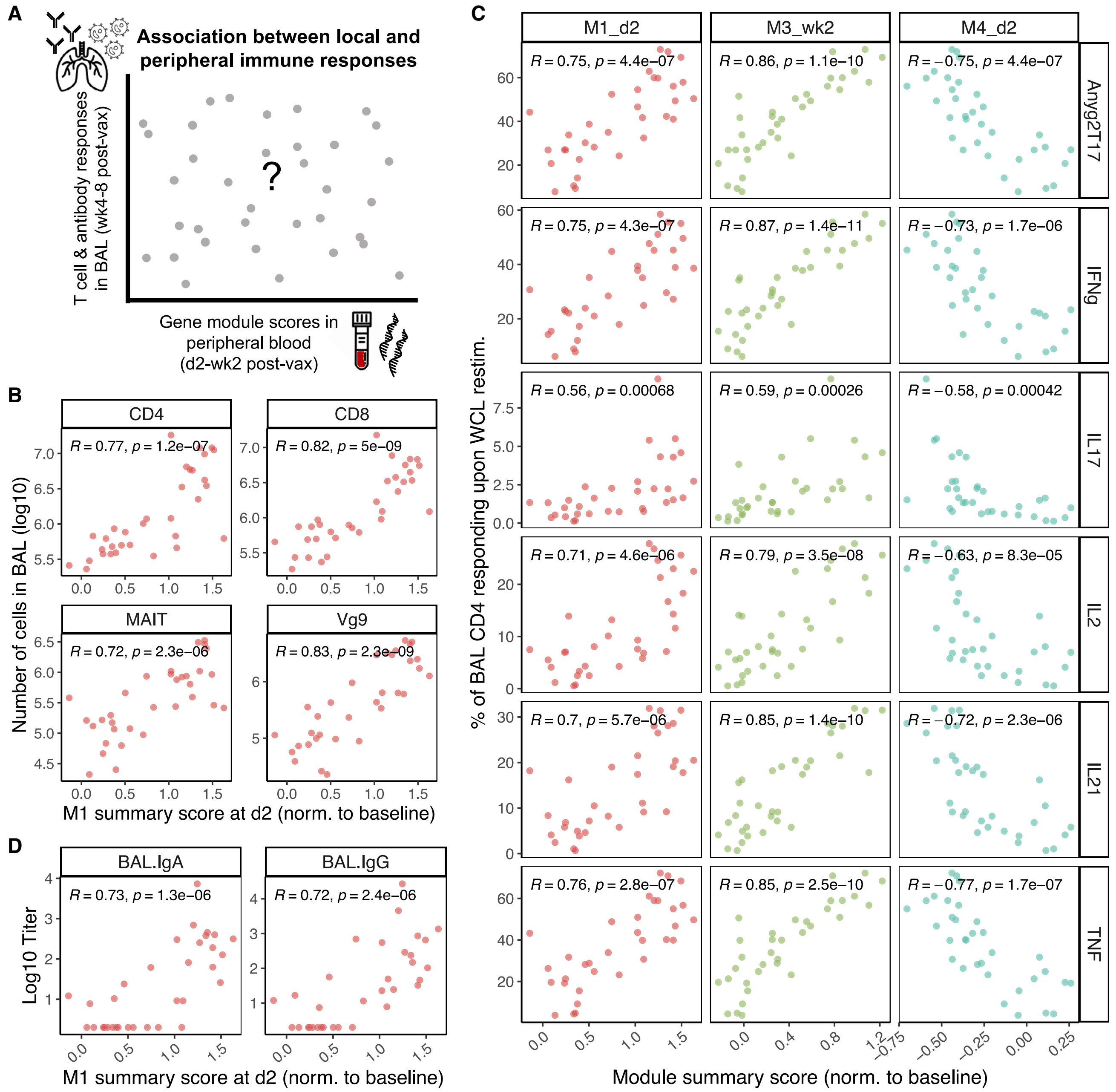

Figure 2. Correlations between module scores in blood and adaptive immune responses in bronchoalveolar lavage (BAL) in the dose cohort (n = 33)

(A) Schematic of correlation between gene module scores in peripheral blood at day 2 or week 2 post-vaccination and T or B cell responses in BAL at 4–8 weeks post-vaccination.

(B) Correlations between module 1 scores at day 2 in blood and T cell counts (log10) at week 4 in BAL.

(C) Correlations between module 1 scores at day 2 post-vaccination, module 3 scores at week 2 post-vaccination, or module 4 scores at day 2 post-vaccination and the frequency of antigen-specific CD4 T cells in BAL at week 8 post-vaccination. Antigen-specific CD4 T cells are defined as the frequency of BAL CD4 T cells expressing a given cytokine or any combination of cytokines (Anyg2T17) upon *ex vivo* restimulation with *Mtb* antigens.

(D) Correlations between module 1 scores at day 2 in blood and IgA or IgG antibody titers (log10) at week 4 in BAL or plasma.

Pearson correlation coefficients and corresponding p values are shown. All module scores were normalized to each animal's baseline.

$p \le 2.3e{-}06$; Figure 2B). Module 1 scores on day 2 were also correlated with numbers of B cells, NK cells, and DCs ($r \ge 0.61$, $p \le 5.3e{-}04$) in BAL but not in macrophages (Figure S2A).

Importantly, module 1 scores on day 2 were associated with the frequency of antigen-responsive CD4 T cells in BAL ($r \ge 0.56$, $p \le 6.8e{-}04$), identified by flow cytometry as CD4 T cells

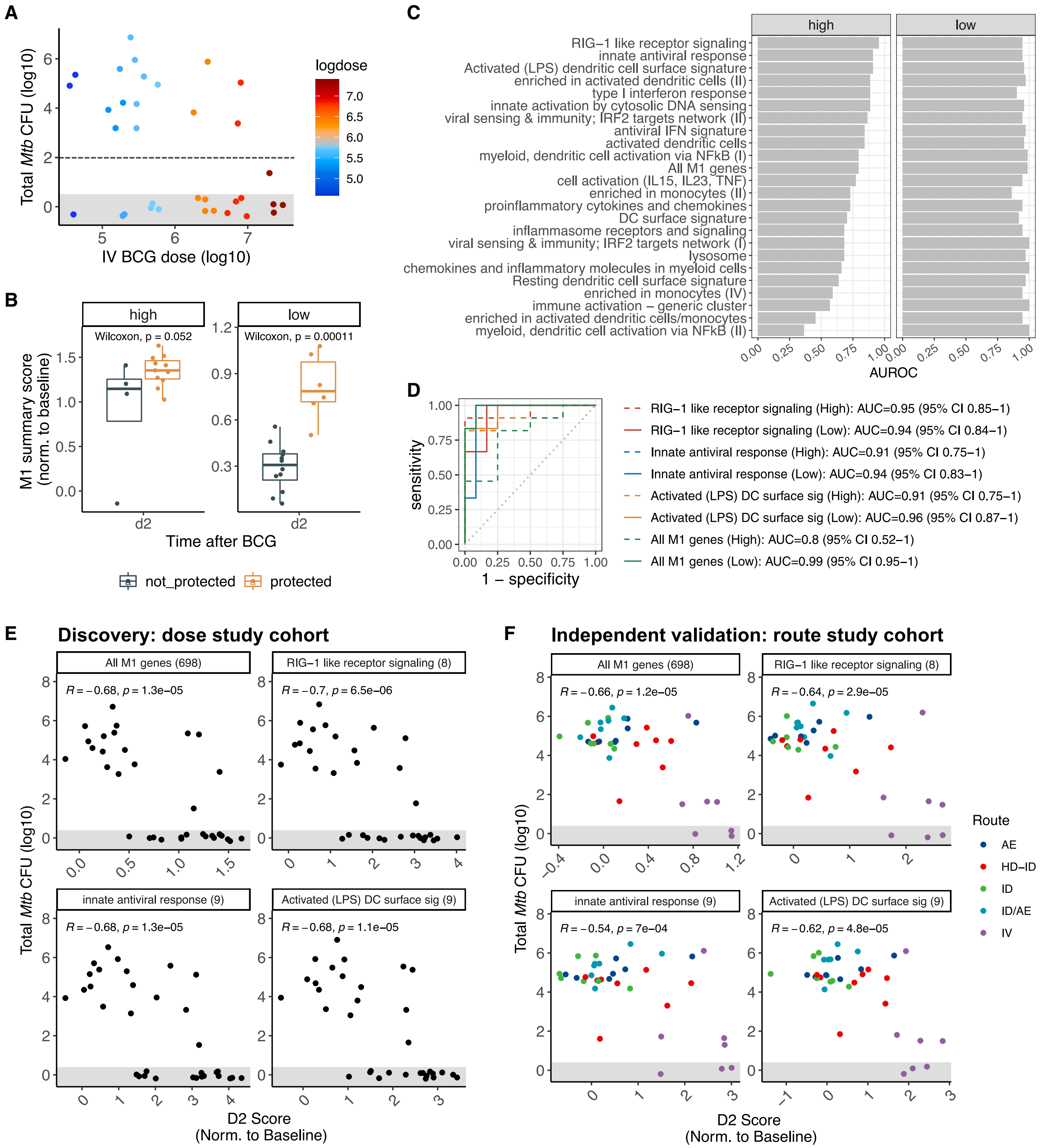

Figure 3. Innate module 1 is a reliable correlate of BCG-induced protection

(A) Total *Mtb* colony-forming units (CFUs) upon necropsy by i.v. BCG dose received. Animals with total *Mtb* CFUs under 10^2 (beneath the horizontal dashed line) are considered protected.

(B) Module 1 scores at day 2 for high-dose (n = 16) or low-dose (n = 18) i.v. BCG recipients, stratified by protection outcome following *Mtb* challenge.

(C) All immune pathways enriched in module 1 and their accuracy at day 2 in predicting protection following challenge among high- or low-dose recipients. AUROC, area under the receiver operating characteristic curve.

(D) ROC curves for the most accurate module 1 sub-pathways in high-dose recipients (dashed lines) and low-dose recipients (solid lines).

(legend continued on next page)

expressing IFNγ, interleukin-2 (IL-2), IL-17, IL-21, and/or tumor necrosis factor (TNF) in response to *Mtb* restimulation 8 weeks post-vaccination (Figure 2C). The frequency of antigen-responsive CD4 T cells in BAL at 8 weeks post-vaccination was also strongly foreshadowed in blood by module 3 scores at week 2 ($r \geq 0.59$, $p \leq 2.6e{-}04$) and module 4 scores on day 2 ($|r| \geq 0.58$, $p \leq 4.2e{-}04$; Figure 2C). However, none of the modules were significantly associated with the frequency of antigen-responsive CD8 T cells in BAL (Figures S2B and S3). Finally, module 1 and module 4 scores at day 2 post-vaccination were strongly correlated with IgA and IgG titers in BAL at 4 weeks post-vaccination ($|r| \geq 0.72$, $p \leq 2.4e{-}06$) (Figure 2D).

We next examined whether any of the seven i.v. BCG-induced modules were associated with protection following *Mtb* challenge, despite having been discovered without considering outcomes following *Mtb* challenge. Eighteen of 34 (53%) macaques were protected against TB (12 high dose and 6 low dose), where protection was defined as fewer than 100 total CFU *Mtb* upon necropsy. A higher dose of i.v. BCG was associated with greater protection (univariate odds ratio for a 10-fold increase in dose = 3.602, p = 0.013), but sterile protection (0 *Mtb* CFUs) was nonetheless observed across all dose groups (Figure 3A). We first assessed potential correlates of protection in the discovery subset of high-dose i.v. BCG recipients. Module 1 scores at day 2 post-vaccination were elevated in high-dose recipients that were protected following *Mtb* challenge compared with those that were not protected (Wilcoxon one-sided p = 0.052; Figure 3B). The difference in module 1 scores at day 2 by protection outcome was conserved and more pronounced in the validation subset of low-dose recipients (Wilcoxon one-sided $p = 1.1e{-}4$; Figure 3B). As module 1 induction varied by dose, we conducted a secondary analysis to test whether its association with protection would remain after adjusting for dose. We modeled module 1 scores at day 2 as a function of dose (as a continuous variable) and protection outcome (STAR Methods). In this non-linear model, module 1 scores at day 2 remained significantly associated with protection outcome even after accounting for dose ($p < 0.001$; Table S4). Although other modules were associated with protection in low-dose i.v. BCG recipients (e.g., module 2 at day 2, module 3 at week 2, and module 4 at day 2), these associations did not hold in high-dose recipients (Figure S4), suggesting that these responses may be important but insufficient for protection.

Given the consistent association of module 1 scores at day 2 with protection following *Mtb* challenge, we further analyzed this module. Module 1 was comprised of 698 genes and enriched for 24 innate immune pathways (FDR < 1%) (Table S2). We hypothesized that, despite being co-correlated, different "sub-pathways" within module 1 may have varying dynamic ranges and therefore may vary in their discriminatory power to predict protection outcomes. To test this, we computed day 2 scores for each enriched sub-pathway and assessed their accuracy in predicting protection following challenge using AUROCs, as well as their correlation with *Mtb* CFUs post-challenge. Indeed, compared with all genes in module 1 which had an AUROC of 0.795 (95% confidence interval [CI], 0.520–1) in high-dose recipients, sub-pathways in module 1 had AUROCs ranging from 0.363 to 0.955 (95% CI: 0.852–1) in high-dose recipients (Figures 3C and 3D; Table S5). In contrast, all sub-pathways had high accuracy in low-dose recipients, with all but one sub-pathway achieving AUROCs over 0.9 (Figures 3C and 3D; Table S5). Strikingly, in addition to predicting binary protection outcome, day 2 scores of module 1 and its parsimonious sub-pathways were strongly negatively correlated ($r \leq -0.59$, $p \leq 2.8e{-}04$) with post-challenge *Mtb* CFUs and with the number of granulomas upon necropsy (Figures 3E and S5A; Table S6). We also observed negative correlations between day 2 scores of module 1 and its sub-pathways with *Mtb* CFUs and granuloma burden in the independent route cohort (Figures 3F and S5B). Arguably, the correlations in the route cohort were driven by the i.v. group. However, when considering only the i.v.-vaccinated animals from the route cohort, module 1 scores at day 2 were still negatively correlated with *Mtb* CFUs ($r = -0.56$, p = 0.19) and granulomas ($r = -0.68$, p = 0.09), where the lack of statistical significance is likely due to the very small sample size (n = 7).

DISCUSSION

Correlates of vaccine-mediated protection against TB will inform the development of more efficacious vaccines. Lung-localized immune responses can be useful for identifying correlates and understanding mechanisms of protection, but BAL samples are more difficult to obtain. Therefore, there is a critical need to identify blood-based correlates that can be more easily measured and scaled. However, TB vaccine studies to date have failed to identify correlates in blood that have sufficient predictive accuracy. In this study, we leveraged a non-human primate model of i.v. BCG vaccination followed by *Mtb* challenge to identify correlates of protection in the blood transcriptome. In one cohort where macaques were randomized to receive varying doses of i.v. BCG, an increasing dose was associated with both stronger transcriptional responses to vaccination and higher levels of protection following *Mtb* challenge. We identified a gene module (module 1), predominantly comprised of innate immune pathways, whose score on day 2 post-vaccination was strongly associated with protection following challenge, even after adjusting for dose. Individual pathways within module 1, consisting of 8–9 genes each, robustly predicted protection outcomes following challenge with AUROCs ≥ 0.9 in both high- and low-dose i.v. BCG recipients. Furthermore, module 1 scores on day 2 post-vaccination were highly correlated with functional T, B, and NK cell responses in the BAL at 4–8 weeks post-vaccination and inversely correlated with the number of granulomas and *Mtb* CFUs in lung following challenge.

(E and F) Correlations between *Mtb* burden (log10 CFUs) upon necropsy and day 2 scores of module 1 or its sub-pathways in the (E) dose study cohort or (F) route study cohort. The number of genes in each set is shown in parentheses to the right of the set name. AE, aerosol; HD-ID, high-dose intradermal; ID, intradermal; ID/AE, intradermal and aerosol; i.v., high-dose intravenous. Pearson correlation coefficients and corresponding p values are shown. All scores were normalized to each animal's baseline. Data points are jittered to reduce overplotting; all points in the shaded gray area represent animals with no detectable CFUs.

Collectively, these findings shed light on the previously underappreciated early innate response to vaccination in peripheral blood, which validated across dose groups and in an independent cohort as an accurate, accessible predictor of protection against TB in macaques.

Compared with module 1 as a whole, several sub-pathways within module 1 demonstrated higher accuracy in predicting protection following challenge across dose groups, including IFN signaling pathways, DC activation pathways, and cytosolic sensing pathways like RLR signaling pathways. Interestingly, in contrast to *Mtb*, BCG may fail to adequately activate such cytosolic sensing pathways due to its sequestration in immature phagosomes, precluding efficient lysosomal degradation and antigen presentation.[26,27] A recent study showed that a dinucleotide agonist of RIG-I increased antigen presentation and IFNβ secretion by BCG-infected macrophages and DCs *in vitro* and enhanced BCG-mediated protection against *Mtb* challenge in a mouse model.[28] i.v. BCG-mediated protection may therefore rely in part on similar mechanisms such as increased activation of RLRs and other innate cytosolic sensing pathways, induction of type 1 IFN signaling, and enhanced antigen presentation, which may collectively contribute to improved priming and expansion of mycobacterial-specific T cells and other protective responses. Future research should investigate the mechanisms by which i.v. BCG achieves this early response to illuminate targets for novel adjuvants to improve BCG efficacy. Moreover, although type 1 IFN signaling has been implicated in other settings as a correlate of risk for TB infection and disease,[29,30] previous studies in mouse and guinea pig models have suggested that type 1 IFNs may play a protective role in the context of BCG-induced immunity against *Mtb* infection,[31] concordant with the present study. Future studies should elucidate the timing- and context-dependent roles of type 1 IFNs in mediating protection or susceptibility, especially in the context of vaccination against TB.

We identified additional elements of the transcriptional response that are likely important for—albeit not sufficiently predictive of—BCG-mediated protection. For instance, module 2, which was also enriched for innate immune cell (monocyte, DC, and neutrophil) pathways, was more transiently upregulated than module 1 but may contribute to priming a subsequent adaptive response. Furthermore, the early downregulation of T and NK cell pathways in module 4 on day 2 post-vaccination may in part reflect lymphocyte homing to secondary lymphoid organs for activation, as corroborated by complete blood count (CBC) data from the previously published route study showing early lymphopenia following vaccination with i.v. or high-dose ID BCG.[12] The ensuing induction of CD4 T cell proliferation pathways in module 3 at weeks 2–4 following vaccination likely also reflects a critical step in establishing a robust lung-resident memory CD4 T cell response. Notably, these transcriptional responses were associated with protection in low-dose, but not high-dose, recipients, suggesting that there may be additional dose-specific protective mechanisms that merit further investigation.

Our findings are critical to consider alongside those from a separate analysis of flow cytometry, Luminex, and CBC data from the same dose cohort (Darrah et al., in press). In that analysis, adaptive responses in BAL were better predictors of protection following challenge than responses in blood. However, in the present study, we found that several modules (M1, M3, and M4) measured at day 2 or week 2 post-vaccination were highly correlated with T cell, NK cell, and antibody responses in BAL at week 4–8 post-vaccination. In addition, our early innate transcriptional signatures in blood predicted protection following *Mtb* challenge with comparable accuracy to the best-performing adaptive BAL features from the separate analysis by Darrah et al. (in press). Therefore, in peripheral blood, transcriptional signatures may provide better correlates of protection than flow cytometry, Luminex, or CBC data and may be adequate surrogates for lung-resident memory responses when BAL samples are unavailable. Of note, our modules were correlated with BAL CD4 T cells expressing all measured cytokines due to high co-correlation among these responses. As Darrah et al. concluded in their manuscript, protection was likely not exclusively driven by a specific Th1 or Th17 cytokine combination but rather a coordinated response involving diverse T cell functions (Darrah et al., in press). Additional studies are needed to mechanistically dissect which specific CD4 T cell phenotypes are functionally required for protection, which may also inform identification of more phenotype-specific blood transcriptional signatures. Furthermore, none of our modules were correlated with mycobacterial-specific CD8 T cell responses in BAL, potentially due to their lower relative frequency compared with mycobacterial-specific CD4 T cells and to their lack of correlation with protection after adjusting for dose (Darrah et al., in press).

Interest in the innate immune response to BCG vaccination has grown in recent years but has been largely directed toward BCG-induced "trained immunity," or *de facto* innate memory.[32] These studies have primarily profiled later post-vaccination time points to demonstrate the longevity of BCG-induced changes in innate cells.[33–37] One study in rhesus macaques of a cytomegalovirus-based TB vaccine identified an innate blood transcriptional signature measured approximately 56 weeks post-vaccination, immediately before *Mtb* challenge, that was associated with post-challenge outcomes.[13] In contrast, to our knowledge, this study is the first to establish that blood transcriptional signatures induced early (within 2 days) post-vaccination are robustly correlated with protection against TB. This phenomenon has been shown for vaccines against other infections, including Ebola virus,[18] influenza,[16] and yellow fever.[24] Furthermore, to date, the field has focused on adaptive responses for identifying correlates of protection against TB,[7,38–40] which are usually profiled weeks or months after vaccination. Therefore, the early response to TB vaccination and its relevance for vaccine-mediated protection remains understudied.

Of the few studies that have examined the blood transcriptome at this early time point following *in vivo* vaccination with BCG or a TB vaccine candidate,[41–44] none have included subsequent lung immune responses or TB outcomes. However, one study found a rapid increase of circulating neutrophils in mice and humans within 24 h of BCG vaccination; in mice, the increase in neutrophil count was required and sufficient for protection against polymicrobial sepsis, indicating that the early response to BCG may also predict non-specific BCG-mediated protection.[42] The neutrophil signal diminished by day 4 post-vaccination, further illustrating the time-restricted nature of

certain responses to vaccination that may be overlooked in studies that only include later time points. Two other studies have found early blood transcriptional responses induced by vaccine candidate M72/AS01 E (in humans)[41] or H56/CAF01 (in mice)[43] that were similar to those we identified. Specifically, at 1–2 days post-vaccination, these studies found upregulation of many of the same innate pathways as those enriched in module 1 of the present study. The study on M72/AS01 E also found downregulation of some of the same NK and T cell pathways that were enriched in our module 4. These overlapping findings in other species vaccinated with distinct subunit vaccines and adjuvants, delivered through non-i.v. routes (intramuscular or subcutaneous), suggest that certain elements of the blood transcriptional response may be conserved across diverse TB vaccine types and routes. It remains to be determined whether the association of these peripheral responses with local responses and protection is also generalizable.

A strength of our study is the systems-based approach that enabled comprehensive profiling of all aspects of the peripheral response to i.v. BCG vaccination rather than only pre-specified targets of interest. Moreover, our analyses included direct comparisons between peripheral transcriptional responses (blood) and local functional responses (BAL), adding to the limited understanding of the relationship between blood and lung biomarkers.[45] Our methodology was also intentionally designed for the challenges of high-dimensional data and a small sample size. Namely, we did not have sufficient statistical power to conduct gene-by-gene multivariate analyses with time, dose, protection, and their interactions as explanatory variables. Instead, we used a "discovery and validation" framework and divided the cohort by dose to minimize the confounding effects of dose while still ensuring that our results were conserved across dose groups. After identifying significant DEGs in high-dose recipients, we conducted all subsequent analyses using gene modules in order to reduce dimensionality and avoid being prone to false discoveries or overfitting. Finally, we further validated the robustness of our findings in an independent cohort of macaques.

Altogether, our results provide new insights into the early innate response to TB vaccination, which we found to be a robust predictor of protection following *Mtb* challenge. While our findings in low-dose i.v. BCG recipients implicate other components that may be important for protection, such as T and NK pathways at later time points, these were not sufficient to explain differing outcomes following challenge in high-dose recipients. Instead, only the strength of select innate responses 2 days post-vaccination, such as the activation of RLR and DC pathways, could accurately predict protection following challenge across dose groups and in an independent cohort of macaques. These findings underscore the critical need for additional research on the early responses required to prime long-lasting protective immunity against TB. This research could enable improved TB vaccines and adjuvant designs and facilitate efficient evaluation of existing and future vaccine candidates.

Limitations of the study

This study has several limitations. First, dose was a strong confounder, affecting both responses to vaccination and protection outcomes. Our methods sought to circumvent this as described above, but there were nonetheless striking differences between low- and high-dose recipients in the protection-associated response. Future studies should identify a single, intermediate dose that confers 50% protection for a more controlled correlates analysis. Next, i.v. BCG-mediated protection in rhesus macaques may differ from protection conferred by other vaccines, through other routes, and for other species. However, the overlap between our findings and those from other non-i.v. BCG studies in mice and humans suggests that some aspects of the protective response may be generalizable and may at least inform strategies to improve BCG efficacy for humans. Our inferences about i.v. BCG-induced immune pathways are limited by the imperfect specificity of gene set annotations; our results should be viewed as hypothesis-generating for future mechanistic studies. Finally, our results were limited by the lower resolution of bulk RNA sequencing, which also limits our ability to determine to what extent changes in module scores reflect changes in cell type proportions versus changes in gene expression. A priority for future research is to perform single-cell-based analyses in order to elucidate cell type-specific responses and identify more granular correlates.

STAR★METHODS

Detailed methods are provided in the online version of this paper and include the following:

- KEY RESOURCES TABLE
- RESOURCE AVAILABILITY
  - ○ Lead contact
  - ○ Materials availability
  - ○ Data and code availability
- EXPERIMENTAL MODEL AND SUBJECT DETAILS
  - ○ Animals
- METHOD DETAILS
  - ○ Study design
  - ○ BCG vaccination
  - ○ Mtb challenge
  - ○ Sample processing and RNA sequencing
  - ○ Analytical approach
- QUANTIFICATION AND STATISTICAL ANALYSIS
  - ○ Transcriptomic data processing
  - ○ Differential gene expression analysis
  - ○ Module analysis
  - ○ Pathway analysis
  - ○ Statistical modeling of modules over time
  - ○ Correlation with lung immune responses
  - ○ Non-linear model adjusting for dose
  - ○ ROC curves

SUPPLEMENTAL INFORMATION

Supplemental information can be found online at https://doi.org/10.1016/j.xcrm.2023.101096.

ACKNOWLEDGMENTS

We thank Hong Zheng, Michele Donato, Madeleine Scott, and Jason Andrews for valuable advice and discussions. This work was supported by the Intramural

CellPress
OPEN ACCESS

Research Program of the Vaccine Research Center at the National Institute of Allergy and Infectious Diseases (NIAID); the Bill and Melinda Gates Foundation (OPP1113682 to P.K. and Aeras and BMGF INV-020435 to J.L.F.); NIAID grants 1U19AI109662 (P.K.), U19AI057229 (P.K.), and 75N93019C00071 (J.L.F.); Department of Defense contracts W81XWH-18-10253 and W81XWH1910235 (P.K.); the Ralph & Marian Falk Medical Research Trust (P.K.); the Knight-Hennessy Scholars Program (Y.E.L.); and the National Science Foundation Graduate Research Fellowship Program (Y.E.L.). The funders had no role in study design, data collection and analysis, decision to publish, or preparation of the manuscript.

AUTHOR CONTRIBUTIONS

Conceptualization, Y.E.L., P.A.D., M.R., J.L.F., R.A.S., and P.K.; methodology and formal analysis, Y.E.L.; investigation, Y.E.L., P.A.D., J.J.Z., M.K., F.L., D.C.D., and P.M.; writing – original draft, Y.E.L., writing – review & editing, Y.E.L., P.A.D., J.J.Z., M.K., F.L., D.C.D., P.M., M.R., J.L.F., R.A.S., and P.K.; supervision, M.R., J.L.F., R.A.S., and P.K.

DECLARATION OF INTERESTS

The authors declare no competing interests.

Received: November 21, 2022
Revised: March 29, 2023
Accepted: June 7, 2023
Published: June 29, 2023

REFERENCES

1. World Health Organization (2021). Global Tuberculosis Report 2021.
2. Mangtani, P., Abubakar, I., Ariti, C., Beynon, R., Pimpin, L., Fine, P.E.M., Rodrigues, L.C., Smith, P.G., Lipman, M., Whiting, P.F., and Sterne, J.A. (2014). Protection by BCG vaccine against tuberculosis: a systematic review of randomized controlled trials. Clin. Infect. Dis. *58*, 470–480.
3. Roy, A., Eisenhut, M., Harris, R.J., Rodrigues, L.C., Sridhar, S., Habermann, S., Snell, L., Mangtani, P., Adetifa, I., Lalvani, A., and Abubakar, I. (2014). Effect of BCG vaccination against Mycobacterium tuberculosis infection in children: systematic review and meta-analysis. BMJ *349*, g4643.
4. Martinez, L., Cords, O., Liu, Q., Acuna-Villaorduna, C., Bonnet, M., Fox, G.J., Carvalho, A.C.C., Chan, P.C., Croda, J., Hill, P.C., et al. (2022). Infant BCG vaccination and risk of pulmonary and extrapulmonary tuberculosis throughout the life course: a systematic review and individual participant data meta-analysis. Lancet Global Health *10*, e1307–e1316.
5. Lewinsohn, D.A., Lewinsohn, D.M., and Scriba, T.J. (2017). Polyfunctional CD4+ T cells as targets for tuberculosis vaccination. Front. Immunol. *8*, 1262.
6. Rodo, M.J., Rozot, V., Nemes, E., Dintwe, O., Hatherill, M., Little, F., and Scriba, T.J. (2019). A comparison of antigen-specific T cell responses induced by six novel tuberculosis vaccine candidates. PLoS Pathog. *15*, e1007643.
7. Bhatt, K., Verma, S., Ellner, J.J., and Salgame, P. (2015). Quest for correlates of protection against tuberculosis. Clin. Vaccine Immunol. *22*, 258–266.
8. Dockrell, H.M., and Smith, S.G. (2017). What have we learnt about BCG vaccination in the last 20 Years? Front. Immunol. *8*, 1134.
9. Tameris, M.D., Hatherill, M., Landry, B.S., Scriba, T.J., Snowden, M.A., Lockhart, S., Shea, J.E., McClain, J.B., Hussey, G.D., Hanekom, W.A., et al. (2013). Safety and efficacy of MVA85A, a new tuberculosis vaccine, in infants previously vaccinated with BCG: a randomised, placebo-controlled phase 2b trial. Lancet *381*, 1021–1028.
10. Kagina, B.M.N., Abel, B., Scriba, T.J., Hughes, E.J., Keyser, A., Soares, A., Gamieldien, H., Sidibana, M., Hatherill, M., Gelderbloem, S., et al. (2010). Specific T cell frequency and cytokine expression profile do not correlate with protection against tuberculosis after Bacillus calmette-guérin vaccination of newborns. Am. J. Respir. Crit. Care Med. *182*, 1073–1079.
11. Fletcher, H.A., Snowden, M.A., Landry, B., Rida, W., Satti, I., Harris, S.A., Matsumiya, M., Tanner, R., O'Shea, M.K., Dheenadhayalan, V., et al. (2016). T-cell activation is an immune correlate of risk in BCG vaccinated infants. Nat. Commun. *7*, 11290.
12. Darrah, P.A., Zeppa, J.J., Maiello, P., Hackney, J.A., Wadsworth, M.H., 2nd, Hughes, T.K., Pokkali, S., Swanson, P.A., 2nd, Grant, N.L., Rodgers, M.A., et al. (2020). Prevention of tuberculosis in macaques after intravenous BCG immunization. Nature *577*, 95–102.
13. Hansen, S.G., Zak, D.E., Xu, G., Ford, J.C., Marshall, E.E., Malouli, D., Gilbride, R.M., Hughes, C.M., Ventura, A.B., Ainslie, E., et al. (2018). Prevention of tuberculosis in rhesus macaques by a cytomegalovirus-based vaccine. Nat. Med. *24*, 130–143.
14. Dijkman, K., Sombroek, C.C., Vervenne, R.A.W., Hofman, S.O., Boot, C., Remarque, E.J., Kocken, C.H.M., Ottenhoff, T.H.M., Kondova, I., Khayum, M.A., et al. (2019). Prevention of tuberculosis infection and disease by local BCG in repeatedly exposed rhesus macaques. Nat. Med. *25*, 255–262.
15. Irvine, E.B., O'Neil, A., Darrah, P.A., Shin, S., Choudhary, A., Li, W., Honnen, W., Mehra, S., Kaushal, D., Gideon, H.P., et al. (2021). Robust IgM responses following intravenous vaccination with Bacille Calmette–Guérin associate with prevention of Mycobacterium tuberculosis infection in macaques. Nat. Immunol. *22*, 1515–1523.
16. Nakaya, H.I., Wrammert, J., Lee, E.K., Racioppi, L., Marie-Kunze, S., Haining, W.N., Means, A.R., Kasturi, S.P., Khan, N., Li, G.M., et al. (2011). Systems biology of vaccination for seasonal influenza in humans. Nat. Immunol. *12*, 786–795.
17. Querec, T.D., Akondy, R.S., Lee, E.K., Cao, W., Nakaya, H.I., Teuwen, D., Pirani, A., Gernert, K., Deng, J., Marzolf, B., et al. (2009). Systems biology approach predicts immunogenicity of the yellow fever vaccine in humans. Nat. Immunol. *10*, 116–125.
18. Rechtien, A., Richert, L., Lorenzo, H., Martrus, G., Hejblum, B., Dahlke, C., Kasonta, R., Zinser, M., Stubbe, H., Matschl, U., et al. (2017). Systems vaccinology identifies an early innate immune signature as a correlate of antibody responses to the Ebola vaccine rVSV-ZEBOV. Cell Rep. *20*, 2251–2261.
19. HIPC-CHI Signatures Project Team; HIPC-I Consortium (2017). Multicohort analysis reveals baseline transcriptional predictors of influenza vaccination responses. Sci. Immunol. *2*, eaal4656.
20. Sweeney, T.E., Braviak, L., Tato, C.M., and Khatri, P. (2016). Genome-wide expression for diagnosis of pulmonary tuberculosis: a multicohort analysis. Lancet Respir. Med. *4*, 213–224.
21. Warsinske, H., Vashisht, R., and Khatri, P. (2019). Host-response-based gene signatures for tuberculosis diagnosis: a systematic comparison of 16 signatures. PLoS Med. *16*, e1002786.
22. Hamada, Y., Penn-Nicholson, A., Krishnan, S., Cirillo, D.M., Matteelli, A., Wyss, R., Denkinger, C.M., Rangaka, M.X., Ruhwald, M., Schumacher, S.G., et al. (2022). Are mRNA Based Transcriptomic Signatures Ready for Diagnosing Tuberculosis in the Clinic? - A Review of Evidence and the Technological Landscape. eBioMedicine, 82.
23. Darrah, P.A., Zeppa, J.J., Wang, C., Irvine, E.B., Buscan, A.N., Rodgers, M.A., Pokkali, S., Hackney, J.A., Kamath, M., White, A.G., et al. (2023). Airway T cells are a correlate of i.v. Bacille Calmette-Guerin-mediated protection against tuberculosis in rhesus macaques. Cell Host Microbe. *31*, 962–977. https://doi.org/10.1016/j.chom.2023.05.006.
24. Li, S., Rouphael, N., Duraisingham, S., Romero-Steiner, S., Presnell, S., Davis, C., Schmidt, D.S., Johnson, S.E., Milton, A., Rajam, G., et al. (2014). Molecular signatures of antibody responses derived from a systems biology study of five human vaccines. Nat. Immunol. *15*, 195–204.
25. McCaffrey, E.F., Donato, M., Keren, L., Chen, Z., Delmastro, A., Fitzpatrick, M.B., Gupta, S., Greenwald, N.F., Baranski, A., Graf, W., et al.

(2022). The immunoregulatory landscape of human tuberculosis granulomas. Nat. Immunol. *23*, 318–329.

26. Jagannath, C., Lindsey, D.R., Dhandayuthapani, S., Xu, Y., Hunter, R.L., Jr., and Eissa, N.T. (2009). Autophagy enhances the efficacy of BCG vaccine by increasing peptide presentation in mouse dendritic cells. Nat. Med. *15*, 267–276.
27. Jagannath, C., Khan, A., and Wang, J. (2018). Chapter 4 - increased immunogenicity through autophagy. In Immunology, M.A. Hayat, ed. (Academic Press), pp. 35–54.
28. Khan, A., Singh, V.K., Mishra, A., Soudani, E., Bakhru, P., Singh, C.R., Zhang, D., Canaday, D.H., Sheri, A., Padmanabhan, S., et al. (2020). NOD2/RIG-I activating inarigivir adjuvant enhances the efficacy of BCG vaccine against tuberculosis in mice. Front. Immunol. *11*, 592333.
29. Donovan, M.L., Schultz, T.E., Duke, T.J., and Blumenthal, A. (2017). Type I interferons in the pathogenesis of tuberculosis: molecular drivers and immunological consequences. Front. Immunol. *8*, 1633.
30. Satti, I., Wittenberg, R.E., Li, S., Harris, S.A., Tanner, R., Cizmeci, D., Jacobs, A., Williams, N., Mulenga, H., Fletcher, H.A., et al. (2022). Inflammation and immune activation are associated with risk of Mycobacterium tuberculosis infection in BCG-vaccinated infants. Nat. Commun. *13*, 6594.
31. Moreira-Teixeira, L., Mayer-Barber, K., Sher, A., and O'Garra, A. (2018). Type I interferons in tuberculosis: foe and occasionally friend. J. Exp. Med. *215*, 1273–1285.
32. Netea, M.G., Domínguez-Andrés, J., Barreiro, L.B., Chavakis, T., Divangahi, M., Fuchs, E., Joosten, L.A.B., van der Meer, J.W.M., Mhlanga, M.M., Mulder, W.J.M., et al. (2020). Defining trained immunity and its role in health and disease. Nat. Rev. Immunol. *20*, 375–388.
33. Kaufmann, E., Sanz, J., Dunn, J.L., Khan, N., Mendonça, L.E., Pacis, A., Tzelepis, F., Pernet, E., Dumaine, A., Grenier, J.C., et al. (2018). BCG educates hematopoietic stem cells to generate protective innate immunity against tuberculosis. Cell *172*, 176–190.e19.
34. Kleinnijenhuis, J., Quintin, J., Preijers, F., Joosten, L.A.B., Jacobs, C., Xavier, R.J., van der Meer, J.W.M., van Crevel, R., and Netea, M.G. (2014). BCG-induced trained immunity in NK cells: role for non-specific protection to infection. Clin. Immunol. *155*, 213–219.
35. Suliman, S., Geldenhuys, H., Johnson, J.L., Hughes, J.E., Smit, E., Murphy, M., Toefy, A., Lerumo, L., Hopley, C., Pienaar, B., et al. (2016). Bacillus calmette–guérin (BCG) Revaccination of Adults with latent Mycobacterium tuberculosis infection induces long-lived BCG-reactive NK cell responses. J. Immunol. *197*, 1100–1110.
36. Kleinnijenhuis, J., Quintin, J., Preijers, F., Joosten, L.A.B., Ifrim, D.C., Saeed, S., Jacobs, C., van Loenhout, J., de Jong, D., Stunnenberg, H.G., et al. (2012). Bacille Calmette-Guérin induces NOD2-dependent nonspecific protection from reinfection via epigenetic reprogramming of monocytes. Proc. Natl. Acad. Sci. USA *109*, 17537–17542.
37. Cirovic, B., de Bree, L.C.J., Groh, L., Blok, B.A., Chan, J., van der Velden, W.J.F.M., Bremmers, M.E.J., van Crevel, R., Händler, K., Picelli, S., et al. (2020). BCG vaccination in humans elicits trained immunity via the hematopoietic progenitor compartment. Cell Host Microbe *28*, 322–334.e5.
38. Scriba, T.J., Netea, M.G., and Ginsberg, A.M. (2020). Key recent advances in TB vaccine development and understanding of protective immune responses against Mycobacterium tuberculosis. Semin. Immunol. *50*, 101431.
39. Divangahi, M., and Behr, M.A. (2018). Cracking the vaccine code in tuberculosis. Am. J. Respir. Crit. Care Med. *197*, 427–432.
40. Dockrell, H.M., and Butkeviciute, E. (2022). Can what have we learnt about BCG vaccination in the last 20 years help us to design a better tuberculosis vaccine? Vaccine *40*, 1525–1533.
41. van den Berg, R.A., De Mot, L., Leroux-Roels, G., Bechtold, V., Clement, F., Coccia, M., Jongert, E., Evans, T.G., Gillard, P., and van der Most, R.G. (2018). Adjuvant-associated peripheral blood mRNA profiles and kinetics induced by the adjuvanted recombinant protein candidate tuberculosis vaccine M72/AS01 in Bacillus calmette-guérin-vaccinated adults. Front. Immunol. *9*, 564.
42. Brook, B., Harbeson, D.J., Shannon, C.P., Cai, B., He, D., Ben-Othman, R., Francis, F., Huang, J., Varankovich, N., Liu, A., et al. (2020). BCG vaccination–induced emergency granulopoiesis provides rapid protection from neonatal sepsis. Sci. Transl. Med. *12*, eaax4517.
43. Santoro, F., Pettini, E., Kazmin, D., Ciabattini, A., Fiorino, F., Gilfillan, G.D., Evenroed, I.M., Andersen, P., Pozzi, G., and Medaglini, D. (2018). Transcriptomics of the vaccine immune response: priming with adjuvant modulates recall innate responses after boosting. Front. Immunol. *9*, 1248.
44. Matsumiya, M., Satti, I., Chomka, A., Harris, S.A., Stockdale, L., Meyer, J., Fletcher, H.A., and McShane, H. (2015). Gene expression and cytokine profile correlate with mycobacterial growth in a human BCG challenge model. J. Infect. Dis. *211*, 1499–1509.
45. Morrison, H., and McShane, H. (2021). Local pulmonary immunological biomarkers in tuberculosis. Front. Immunol. *12*, 640916.
46. Martin, M. (2011). Cutadapt removes adapter sequences from high-throughput sequencing reads. EMBnet. j. *17*, 10.
47. Bray, N.L., Pimentel, H., Melsted, P., and Pachter, L. (2016). Near-optimal probabilistic RNA-seq quantification. Nat. Biotechnol. *34*, 525–527.
48. Love, M.I., Huber, W., and Anders, S. (2014). Moderated estimation of fold change and dispersion for RNA-seq data with DESeq2. Genome Biol. *15*, 550.
49. Langfelder, P., and Horvath, S. (2008). WGCNA: an R package for weighted correlation network analysis. BMC Bioinf. *9*, 559.
50. Bioinformatics, B., FastQC.
51. Durinck, S., Spellman, P.T., Birney, E., and Huber, W. (2009). Mapping identifiers for the integration of genomic datasets with the R/Bioconductor package biomaRt. Nat. Protoc. *4*, 1184–1191.

STAR★METHODS

KEY RESOURCES TABLE

REAGENT or RESOURCE	SOURCE	IDENTIFIER
**Deposited data**		
Raw and processed RNA-sequencing data from dose cohort	This paper; Darrah et al. *Cell Host & Microbe* 2023	GEO: GSE218270
Raw and processed RNA-sequencing data from route cohort	This paper, Darrah et al. *Nature* 2020	GEO: GSE218157
**Software and algorithms**		
Code for analysis and visualization	This paper	https://github.com/Khatri-Lab/bcg_transcriptome
CutAdapt	Martin et al.[46]	https://cutadapt.readthedocs.io/en/stable/
FastQC	Babraham Bioinformatics	https://www.bioinformatics.babraham.ac.uk/projects/fastqc/
Kallisto	Bray et al.[47]	https://pachterlab.github.io/kallisto/
R 4.1.1	The R Project for Statistical Computing	r-project.org
DESeq2 1.34.0	Love et al.[48]	https://bioconductor.org/packages/release/bioc/html/DESeq2.html
WGCNA	Langfelder & Horvath[49]	https://horvath.genetics.ucla.edu/html/CoexpressionNetwork/Rpackages/WGCNA/
Blood transcription modules (BTMs)	Li et al.[24]	https://github.com/shuzhao-li/BTM

RESOURCE AVAILABILITY

Lead contact

Further information and requests for resources and reagents should be directed to and will be fulfilled by the lead contact, Purvesh Khatri (pkhatri@stanford.edu).

Materials availability

This study did not generate new reagents.

Data and code availability

Bulk RNA-seq data have been deposited at GEO and are publicly available as of the date of publication. Accession numbers are listed in the key resources table. All original code has been deposited at https://github.com/Khatri-Lab/bcg_transcriptome and is publicly available as of the date of publication. Any additional information required to reanalyze the data reported in this paper is available from the lead contact upon request.

EXPERIMENTAL MODEL AND SUBJECT DETAILS

Animals

All animals were adult Indian-origin rhesus macaques (*Macaca multta*), sourced from an outbred colony, housed in same-sex pairs. Thirty-four animals (16 male, 18 female; median age 4.4 years) from the "dose" cohort and thirty-six animals (23 male, 13 female; median age 3.2 years) from the "route" cohort were included. Animal work was approved by the Institutional Care & Use Committees of the respective institutions (NIH Vaccine Research Center, Bioqual, Inc., and the University of Pittsburgh). Macaques were housed and cared for in facilities accredited by the American Association for Accreditation of Laboratory Animal Care (AAALAC) and in accordance with the guidelines outlined by the Animal Welfare Act and Regulation (USDA) and the Guide for the Care & Use of Laboratory Animals, eighth Edition (NIH). Macaques were housed at Bioqual, Inc. during the vaccination phase and transferred to the University of Pittsburgh (ABSL-3) during the challenge phase. Macaques were monitored for physical health, food consumption, body weight, temperature (rectal probe), complete blood counts, and serum chemistries. Macaques were not involved in previous procedures and were drug-naïve.

METHOD DETAILS

Study design

Thirty-four macaques (the dose cohort) were randomized into six vaccine groups to receive varying doses of IV BCG (4.5–7.5 log10 CFU, in half-log increments). Whole blood was collected for bulk RNA sequencing at baseline (four weeks before vaccination) and two days, two weeks, four weeks, and twelve weeks following vaccination. Macaques were challenged 5–6 months after vaccination with a low dose of *Mtb* and euthanized 12 weeks later, or at the humane endpoint, for analysis of disease burden (Darrah et al., in press). For the previously published route cohort,[12] thirty-six macaques were randomized to receive BCG vaccination through different routes: aerosol (AE, n = 7), intradermal (ID, n = 7), high-dose intradermal (HD-ID, n = 8), combined aerosol and intradermal (AE/ID, n = 7), and intravenous (IV, n = 7). IV-vaccinated macaques in the route cohort received 5×10^7 CFU of BCG. Macaques were challenged with *Mtb* 6–10 months following BCG vaccination and euthanized 11–15 weeks following challenge or at humane endpoint. For the route cohort, whole blood samples were collected for RNA sequencing at baseline and two days, two weeks, and twelve weeks following vaccination. Randomization for both studies was performed based on birth colony, gender, and pre-vaccination CD4 T cell responses to PPD in BAL.

BCG vaccination

BCG Danish strain 1331 (Statens Serum Institute), expanded and cryopreserved by Aeras (now IAVI), was serially diluted in cold PBS with 0.05% tyloxapol (Sigma-Aldrich) and administered intravenously into the saphenous vein (2mL) for IV BCG recipients (varying doses in the dose cohort, 5×10^7 CFU in the route cohort). For the other groups in the route cohort, vaccination was as follows: for the standard ID group, 5×10^5 CFU of BCG was injected in the left upper arm ($5x10^5$); for HD-ID, 5×10^7 CFU of BCG was split between both upper arms (with a volume of 100-200μL per site for both ID groups); for AE, 5×10^7 CFU of BCG in 2mL volume was administered using a pediatric mask attached to a Pari eFlow nebulizer (PARI Pharma GmgH) that delivered 4uM particles into the lung for the AE group; for AE/ID, 5×10^7 CFU AE and 5×10^5 CFU ID in left arm were delivered simultaneously. All listed doses are nominal; empirical CFUs for each vaccine regimen were quantified immediately after vaccination and are available in the primary study manuscripts[12] (Darrah et al., in press) as well as in the metadata tables accompanying the GEO datasets.

Mtb challenge

Macaques were challenged via bronchoscope with an average of 12 CFU (range 4–17 CFU) of *Mtb* Erdman in 2mL PBS. Doses in this range yield similar degrees of TB disease in unvaccinated rhesus macaques[12] (Darrah et al., in press). Macaques were monitored for appetite, behavior, body weight, cough. Humane endpoint criteria were based on these signs, as well as serial erythrocyte sedimentation rate, *Mtb* growth from gastric aspirate, and PET CT.

Sample processing and RNA sequencing

Whole blood was collected in PAXgene blood RNA tubes (Qiagen) and stored at −80C for batch processing at end of study. RNA was extracted using the PAXgene Blood RNA Tube kit (PreAnalytiX) as instructed. Globin mRNA was removed using GLOBINclear Kit (Life Technologies) and remaining mRNA concentration and quality was measured on an Agilent Bioanalyzer using an Agilent nano 6000 kit. Illumina-ready libraries were generated using NEBNext Ultra II RNA Preparation reagents (New England BioLabs). The barcoded Illumina-ready libraries were sequenced using paired-end 151-base protocol on either a NovaSeq 6000 (Illumina) or a HiSeq 4000 (Illumina).

Analytical approach

Due to the limited sample size and high-dimensional nature of the data, we were underpowered to adjust for dose and its interactions with time and protection outcome in multivariate models. Therefore, we took a training-and-validation approach. We dichotomized IV BCG dose at the log-scale midpoint of 10^6 CFU into a binary variable of high or low dose for our main analyses. This threshold enabled representation of protected and non-protected outcomes in both groups. We performed discovery analyses in high-dose recipients and sought to validate our findings in low-dose recipients to ensure generalizability by dose. Specifically, we first identified vaccine-induced genes and modules in high-dose recipients without considering outcomes following challenge. We then assessed which modules were associated with protection following challenge and validated putative correlates in low-dose recipients. We sought to further validate our findings in the independent route cohort.[12]

After module discovery, we performed additional analyses with dose as a continuous variable (see below, "Non-linear model adjusting for dose"). We also examined associations between blood transcriptional responses and local immune responses in the lung (BAL) measured using multi-parameter flow cytometry and Luminex assays as outlined in a separate manuscript (Darrah et al., in press).

QUANTIFICATION AND STATISTICAL ANALYSIS

Transcriptomic data processing

We trimmed reads with Cutadapt[46] and performed quality control checks with FastQC[50] before aligning to the *Macaca mulatta* genome (mmul_10) using kallisto.[47] We removed one sample from the route cohort with fewer than 2000 total sequences. We

CellPress
OPEN ACCESS

removed genes in the globin family, genes that were unannotated, and genes with fewer than 5 reads in over two-thirds of all samples. We performed hierarchical clustering to check for outliers and removed one sample that clustered separately from all other samples. This resulted in 167 samples from the dose discovery cohort and 144 samples from the route validation cohort for analysis. We applied DESeq's variance stabilizing transformation to generate normalized expression values for visualization. All data processing was performed independently for the dose study and route study cohorts.

Time after BCG	Baseline	2 days	2 weeks	4 weeks	12 weeks	Total
Number of samples (dose study cohort)	33	33	34	33	34	167
Number of samples (route study cohort)	36	36	36	NA	36	144

Differential gene expression analysis

We identified genes that were differentially expressed at each timepoint following vaccination, relative to baseline, in high dose IV-BCG recipients from the "dose" discovery cohort. Using DESeq2,[48] we employed a multi-factor design with animal ID to account for between-subject variability at baseline. We utilized approximate posterior estimation for $\log_2$ fold change (LFC) shrinkage[46] and applied significance thresholds of $|LFC| \geq 1.5$ and adjusted p value ≤ 0.1.

Module analysis

To identify coordinated gene modules induced by BCG vaccination, we performed weighted gene correlation network analysis (WGCNA)[49] with the union of genes that were differentially expressed at one or more timepoints following vaccination. We chose the soft thresholding power of 9, which achieved a scale-free topology R^2 of 0.85. We set a minimum module size of 50 genes and merged similar modules whose distance was less than 0.2. One hundred and ninety-one genes remained unassigned. We summarized the expression pattern of each of the final seven modules using a summary score computed as the geometric mean expression of all genes in each module, normalized to each animal's baseline. For one macaque that was missing a baseline sample, we imputed baseline gene expression values using the median baseline expression across all other animals.

Pathway analysis

To determine the immune pathways enriched in each module, we performed over-representation analysis using the hypergeometric test, with blood transcription modules (BTMs) as the reference gene set database.[24] Human gene names in the BTM database were converted to rhesus macaque Ensembl gene IDs using *biomaRt*.[51] We applied a significance threshold of FDR $\leq$ 0.01 and excluded "to be annotated" (TBA) BTMs. For sub-module BTM "scores", we computed the geometric mean expression of module genes in each significantly enriched BTM, normalized to baseline for each animal.

Statistical modeling of modules over time

To statistically test and compare changes in module expression over time following vaccination across dose groups, we utilized generalized estimating equations (GEE), which is an extension of generalized linear models that accounts for within-subject (animal) correlation for repeated measures data. We fit separate GEE models for each module and dose group. Baseline, day 2, week 2, and week 4 timepoints were included. Module summary score was the response variable. Explanatory variables were time (in days), $time^2$, and $time^3$. We included quadratic and cubic terms based on empirically observed trends in module activity over time and based on a goodness-of-fit metric (quasi-likelihood under the independence model criterion or QIC). We also used QIC to select the optimal correlation matrix, preferring simpler models (fewer calibration parameters to estimate) when QICs were comparable. We computed 95% confidence intervals and p values using robust standard errors. We considered changes in module scores over time to be similar between high and low dose recipients if both of the below criteria were met.

1) Coefficients for at least one of the time terms were significant at p $\leq$ 0.05 for low-dose recipients, indicating significant changes in module scores over time among low-dose recipients.
2) Coefficients for all of the time terms were in the same direction (positive/negative) for low-dose recipients as for high-dose recipients, indicating that even when changes over time were greater in magnitude among high-dose recipients, the overall trend and directionality of change over time was consistent across dose groups.

To assess whether changes in module scores over time were similar between high-dose IV BCG recipients in the dose and route cohorts, we combined the data from both cohorts and fit a GEE model for each module, this time also including cohort as an explanatory variable to account for cohort-specific differences at baseline. (We could not fit a separate GEE model for IV BCG recipients in the route cohort because there were too few animals and no week 4 timepoint for the route cohort.) We considered changes in module scores over time to be similar between high-dose IV BCG recipients in the dose and route cohorts if coefficients for the time terms in the combined model were in the same direction as the respective coefficients from the model fit on only the dose cohort animals.

Correlation with lung immune responses

Immune responses in the bronchoalveolar lavage (BAL) after IV BCG vaccination were measured as described separately (Darrah et al., in press). In the present study, we focused on B- and T-cell responses in BAL that were previously shown in the route cohort to be higher after IV BCG vaccination, compared to AE or ID BCG at the same dose. We compared module expression in blood with the following immune markers in BAL: IgA and IgG antibody titers against *Mtb* whole cell lysate; CD4, CD8, Vγ9 γδ, and MAIT T cell counts; and antigen-specific memory CD4 and CD8 T cell responses (production of IFNγ, IL-2, IL-17, IL-21, TNF upon restimulation with *Mtb* purified protein derivative (PPD)).

Non-linear model adjusting for dose

To determine whether module expression was still correlated with protection after adjusting for dose, we fit a non-linear least squares model using a generalized logistic function akin to a dose-response curve with the following formula:

$$S = A + \beta_A + \frac{K - (A+\beta_A)}{\left(1+e^{-B(dose-M)}\right)}$$

Where S is the module score at a given time (normalized to baseline), A is the lower asymptote which varies depending on protection outcome by a fitted coefficient of β_A, K is the upper asymptote, B is the growth rate, and M is the dose midpoint or curve inflection point. We report β_A and its corresponding p value as an indicator of the dose-adjusted estimate for how module expression differs between animals that were or were not protected following challenge.

ROC curves

To compare the accuracy of peripheral transcriptional responses versus local immune responses to vaccination in predicting protection following challenge, we computed area under the receiver operating characteristic (ROC) curves (AUROCs). For blood transcriptional responses, we used the geometric mean scores defined above for modules and BTMs within a module. We computed separate AUROCs for high dose and low dose recipients in order to assess generalizability by dose.

Cell Reports Medicine, Volume *4*

Supplemental information

Blood transcriptional correlates of BCG-induced protection against tuberculosis in rhesus macaques

Yiran E. Liu, Patricia A. Darrah, Joseph J. Zeppa, Megha Kamath, Farida Laboune, Daniel C. Douek, Pauline Maiello, Mario Roederer, JoAnne L. Flynn, Robert A. Seder, and Purvesh Khatri

Table S4. Results of a non-linear model of module 1 scores on day 2 as a function of dose and protection outcome. To assess whether module 1 scores on day 2 were still associated with protection after adjusting for dose, we fit a non-linear least squares model using a generalized logistic function, akin to a dose-response curve with protection outcome as a covariate (see **STAR Methods:** Non-linear modeling adjusting for dose). The parameter β_A indicates how module 1 scores on day 2 differed by protection outcome, after accounting for dose.

**Variable**	**Description**	**Estimate**	**P-value**
A	lower asymptote	0.196	0.237
β_A	change if protected	0.663	<0.001
K	upper asymptote	1.441	<0.001
B	growth rate	2.731	0.142
M	dose midpoint	6.375	<0.001

Table S5. AUCs of all M1 genes or M1 sub-pathways in predicting protection following challenge. AUCs and confidence intervals were computed separately for high- and low-dose recipients. Related to Figure 3c-d.

Module or sub-pathway	AUC	CI_lower	CI_upper	Dose
Activated (LPS) dendritic cell surface signature (S11)	0.909	0.754	1.000	high
activated dendritic cells (M67)	0.841	0.626	1.000	high
All M1 genes	0.795	0.520	1.000	high
antiviral IFN signature (M75)	0.841	0.628	1.000	high
cell activation (IL15, IL23, TNF) (M24)	0.773	0.493	1.000	high
chemokines and inflammatory molecules in myeloid cells (M86.0)	0.659	0.221	1.000	high
DC surface signature (S5)	0.705	0.362	1.000	high
enriched in activated dendritic cells (II) (M165)	0.886	0.709	1.000	high
enriched in activated dendritic cells/monocytes (M64)	0.455	0.067	0.843	high
enriched in monocytes (II) (M11.0)	0.727	0.382	1.000	high
enriched in monocytes (IV) (M118.0)	0.591	0.187	0.995	high
immune activation - generic cluster (M37.0)	0.568	0.109	1.000	high
inflammasome receptors and signaling (M53)	0.682	0.355	1.000	high
innate activation by cytosolic DNA sensing (M13)	0.886	0.706	1.000	high
innate antiviral response (M150)	0.909	0.754	1.000	high
lysosome (M209)	0.682	0.298	1.000	high
myeloid, dendritic cell activation via NFkB (I) (M43.0)	0.795	0.509	1.000	high
myeloid, dendritic cell activation via NFkB (II) (M43.1)	0.364	0.000	0.798	high
proinflammatory cytokines and chemokines (M29)	0.727	0.416	1.000	high
Resting dendritic cell surface signature (S10)	0.636	0.189	1.000	high
RIG-1 like receptor signaling (M68)	0.955	0.852	1.000	high
type I interferon response (M127)	0.886	0.709	1.000	high
viral sensing & immunity; IRF2 targets network (I) (M111.0)	0.682	0.337	1.000	high
viral sensing & immunity; IRF2 targets network (II) (M111.1)	0.864	0.672	1.000	high
Activated (LPS) dendritic cell surface signature (S11)	0.958	0.866	1.000	low
activated dendritic cells (M67)	0.958	0.877	1.000	low
All M1 genes	0.986	0.948	1.000	low
antiviral IFN signature (M75)	0.972	0.908	1.000	low
cell activation (IL15, IL23, TNF) (M24)	0.944	0.826	1.000	low
chemokines and inflammatory molecules in myeloid cells (M86.0)	1.000	1.000	1.000	low
DC surface signature (S5)	0.917	0.769	1.000	low
enriched in activated dendritic cells (II) (M165)	0.972	0.907	1.000	low
enriched in activated dendritic cells/monocytes (M64)	0.944	0.844	1.000	low
enriched in monocytes (II) (M11.0)	0.861	0.586	1.000	low
enriched in monocytes (IV) (M118.0)	0.944	0.826	1.000	low
immune activation - generic cluster (M37.0)	1.000	1.000	1.000	low

inflammasome receptors and signaling (M53)	0.944	0.826	1.000	low
innate activation by cytosolic DNA sensing (M13)	0.958	0.877	1.000	low
innate antiviral response (M150)	0.944	0.830	1.000	low
lysosome (M209)	0.972	0.907	1.000	low
myeloid, dendritic cell activation via NFkB (I) (M43.0)	0.986	0.948	1.000	low
myeloid, dendritic cell activation via NFkB (II) (M43.1)	1.000	1.000	1.000	low
proinflammatory cytokines and chemokines (M29)	0.944	0.844	1.000	low
Resting dendritic cell surface signature (S10)	0.972	0.908	1.000	low
RIG-1 like receptor signaling (M68)	0.944	0.844	1.000	low
type I interferon response (M127)	0.903	0.762	1.000	low
viral sensing & immunity; IRF2 targets network (I) (M111.0)	1.000	1.000	1.000	low
viral sensing & immunity; IRF2 targets network (II) (M111.1)	0.944	0.843	1.000	low
Activated (LPS) dendritic cell surface signature (S11)	0.923	0.836	1.000	combined
activated dendritic cells (M67)	0.908	0.808	1.000	combined
All M1 genes	0.897	0.782	1.000	combined
antiviral IFN signature (M75)	0.908	0.806	1.000	combined
cell activation (IL15, IL23, TNF) (M24)	0.882	0.761	1.000	combined
chemokines and inflammatory molecules in myeloid cells (M86.0)	0.886	0.748	1.000	combined
DC surface signature (S5)	0.868	0.736	1.000	combined
enriched in activated dendritic cells (II) (M165)	0.923	0.835	1.000	combined
enriched in activated dendritic cells/monocytes (M64)	0.849	0.699	1.000	combined
enriched in monocytes (II) (M11.0)	0.853	0.708	0.998	combined
enriched in monocytes (IV) (M118.0)	0.857	0.709	1.000	combined
immune activation - generic cluster (M37.0)	0.871	0.720	1.000	combined
inflammasome receptors and signaling (M53)	0.875	0.745	1.000	combined
innate activation by cytosolic DNA sensing (M13)	0.908	0.808	1.000	combined
innate antiviral response (M150)	0.923	0.835	1.000	combined
lysosome (M209)	0.882	0.751	1.000	combined
myeloid, dendritic cell activation via NFkB (I) (M43.0)	0.897	0.781	1.000	combined
myeloid, dendritic cell activation via NFkB (II) (M43.1)	0.879	0.736	1.000	combined
proinflammatory cytokines and chemokines (M29)	0.882	0.760	1.000	combined
Resting dendritic cell surface signature (S10)	0.871	0.729	1.000	combined
RIG-1 like receptor signaling (M68)	0.923	0.837	1.000	combined
type I interferon response (M127)	0.904	0.806	1.000	combined
viral sensing & immunity; IRF2 targets network (I) (M111.0)	0.882	0.749	1.000	combined
viral sensing & immunity; IRF2 targets network (II) (M111.1)	0.904	0.803	1.000	combined

Table S6. Correlation between M1 sub-pathways and total Mtb CFU (log10) post-challenge. Pearson correlation coefficients and corresponding p-values are shown. Related to Figure 3e.

**Module or sub-pathway**	**Cor.Coef**	**P.value**
Activated (LPS) dendritic cell surface signature (S11)	-0.684	1.12E-05
activated dendritic cells (M67)	-0.680	1.36E-05
All M1 genes	-0.680	1.33E-05
antiviral IFN signature (M75)	-0.659	3.04E-05
cell activation (IL15, IL23, TNF) (M24)	-0.625	9.94E-05
chemokines and inflammatory molecules in myeloid cells (M86.0)	-0.647	4.67E-05
DC surface signature (S5)	-0.612	1.55E-04
enriched in activated dendritic cells (II) (M165)	-0.671	1.89E-05
enriched in activated dendritic cells/monocytes (M64)	-0.617	1.29E-04
enriched in monocytes (II) (M11.0)	-0.642	5.69E-05
enriched in monocytes (IV) (M118.0)	-0.615	1.41E-04
immune activation - generic cluster (M37.0)	-0.647	4.79E-05
inflammasome receptors and signaling (M53)	-0.668	2.16E-05
innate activation by cytosolic DNA sensing (M13)	-0.665	2.44E-05
innate antiviral response (M150)	-0.681	1.28E-05
lysosome (M209)	-0.688	9.65E-06
myeloid, dendritic cell activation via NFkB (I) (M43.0)	-0.621	1.16E-04
myeloid, dendritic cell activation via NFkB (II) (M43.1)	-0.608	1.75E-04
proinflammatory cytokines and chemokines (M29)	-0.604	1.95E-04
Resting dendritic cell surface signature (S10)	-0.604	1.97E-04
RIG-1 like receptor signaling (M68)	-0.697	6.49E-06
type I interferon response (M127)	-0.654	3.60E-05
viral sensing & immunity; IRF2 targets network (I) (M111.0)	-0.621	1.15E-04
viral sensing & immunity; IRF2 targets network (II) (M111.1)	-0.658	3.11E-05

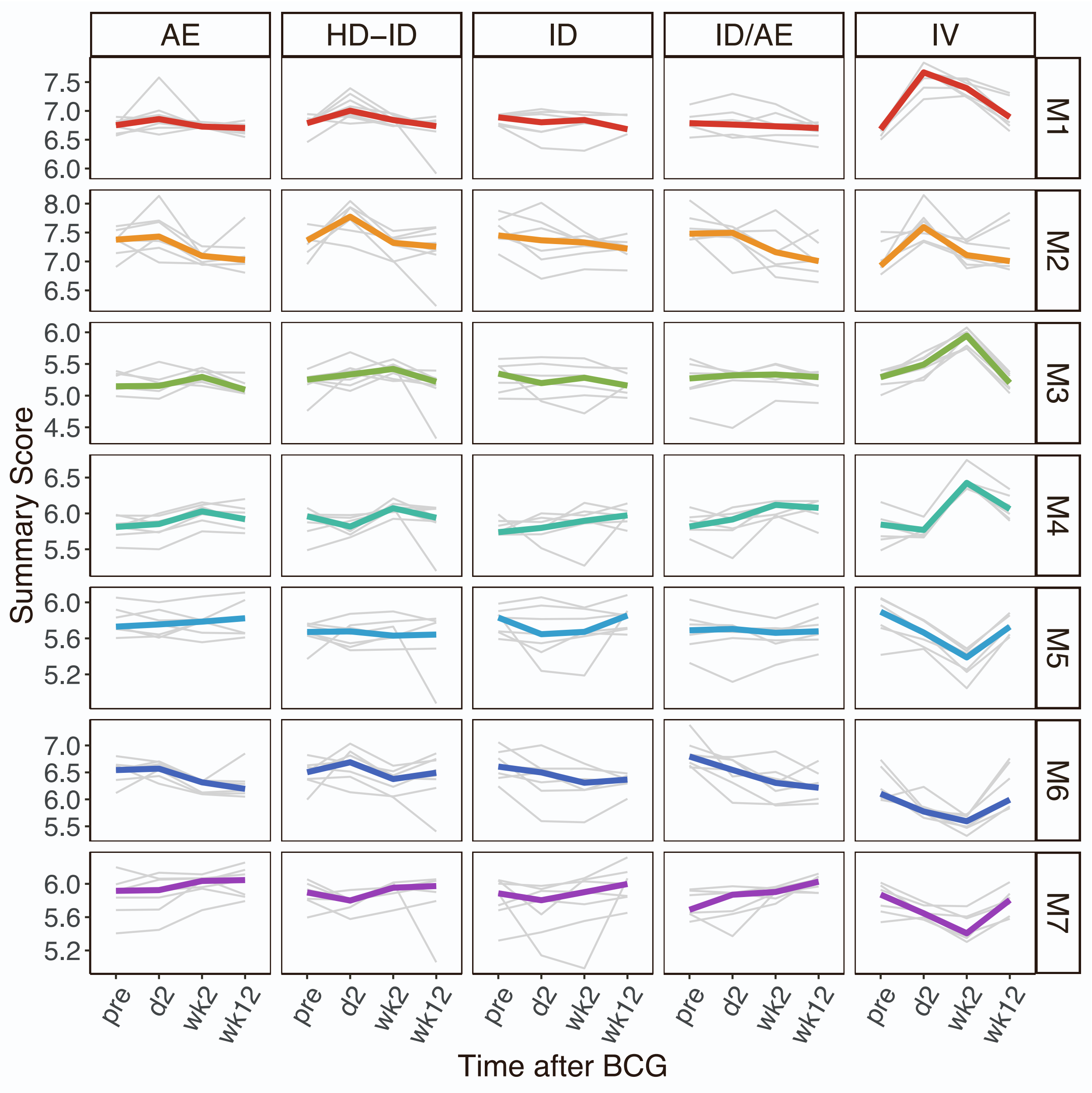

Figure S1. Module activity in macaques from the route study cohort. Summary scores of all seven modules over time following vaccination in the route study cohort, where macaques were BCG-vaccinated via different routes. AE, aerosol; HD-ID, high-dose intradermal; ID, intradermal; ID/AE, intradermal and aerosol; IV, high-dose intravenous. Shown are module scores for individual macaques (thin grey lines) and the median per group (thick colored lines). Related to Figure 1e-f.

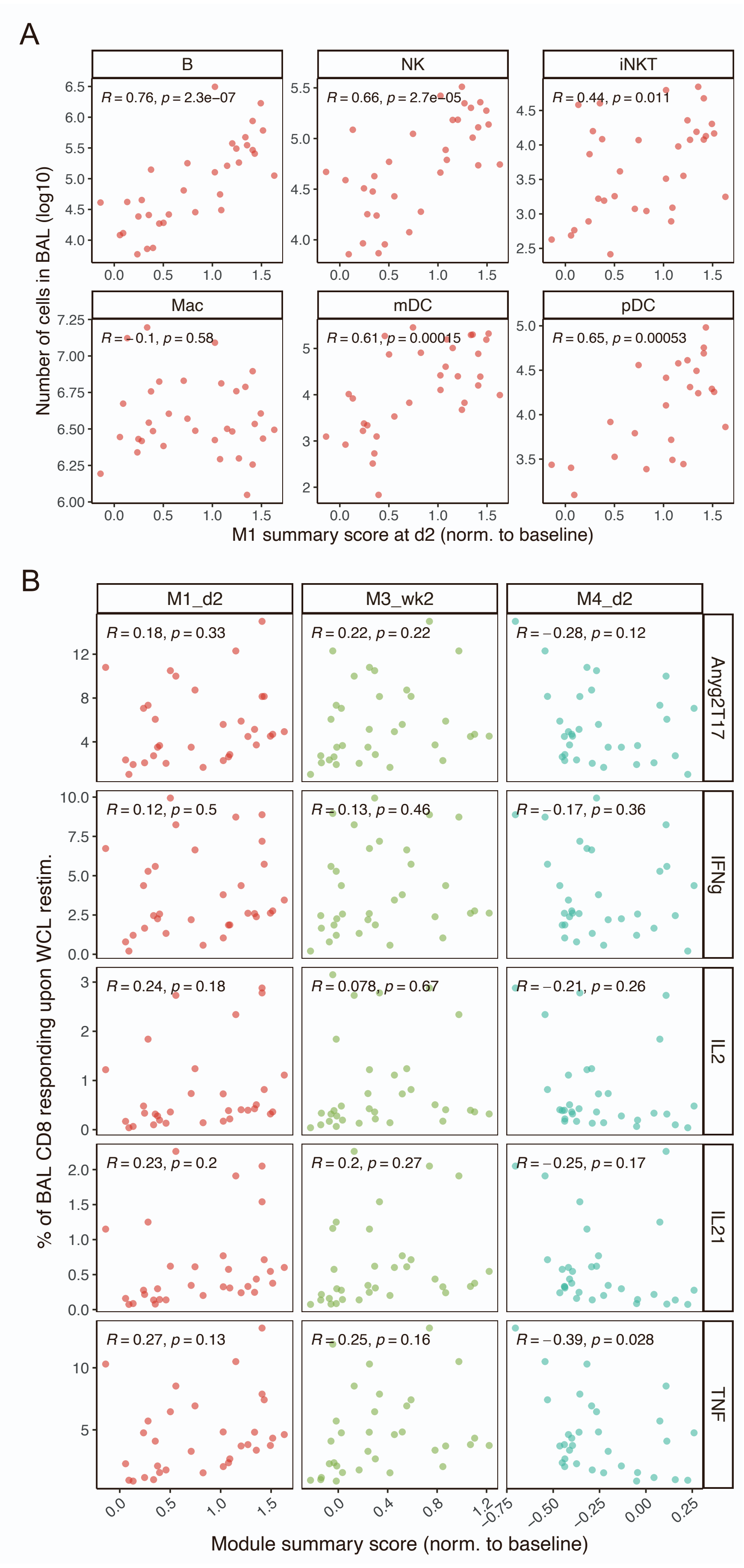
A
B
NK
iNKT
Mac
mDC
pDC
R = 0.76, p = 2.3e−07
R = 0.66, p = 2.7e−05
R = 0.44, p = 0.011
R = −0.1, p = 0.58
R = 0.61, p = 0.00015
R = 0.65, p = 0.00053
Number of cells in BAL (log10)
M1 summary score at d2 (norm. to baseline)
B
M1_d2
M3_wk2
M4_d2
Anyg2T17
IFNg
IL2
IL21
TNF
R = 0.18, p = 0.33
R = 0.22, p = 0.22
R = −0.28, p = 0.12
R = 0.12, p = 0.5
R = 0.13, p = 0.46
R = −0.17, p = 0.36
R = 0.24, p = 0.18
R = 0.078, p = 0.67
R = −0.21, p = 0.26
R = 0.23, p = 0.2
R = 0.2, p = 0.27
R = −0.25, p = 0.17
R = 0.27, p = 0.13
R = 0.25, p = 0.16
R = −0.39, p = 0.028
% of BAL CD8 responding upon WCL restim.
Module summary score (norm. to baseline)

Figure S2. Correlations between module scores in blood and immune responses in BAL. A) Correlations between module 1 scores at day 2 post-vaccination and number (log10) of B cells, NK cells, iNKT cells, macrophages, mDCs, or pDCs in BAL. All BAL cell counts were measured four weeks post-vaccination except NK cell counts which were measured eight weeks post-vaccination. pDC cell count measurements were missing for nine (26%) animals. B) Correlations between module 1 scores at day 2 post-vaccination, module 3 scores at week 2 post-vaccination, or module 4 scores at day 2 post-vaccination and the frequency of antigen-specific CD8 T cells in BAL at week 8 post-vaccination. Antigen-specific CD8 T cells are defined as the frequency of BAL CD8 T cells expressing a given cytokine or any combination of cytokines (Anyg2T17) upon *ex vivo* restimulation with *Mtb* whole cell lysate. Pearson correlation coefficients and corresponding p-values are shown. All module scores were normalized to each animal's baseline. Related to Figure 2.

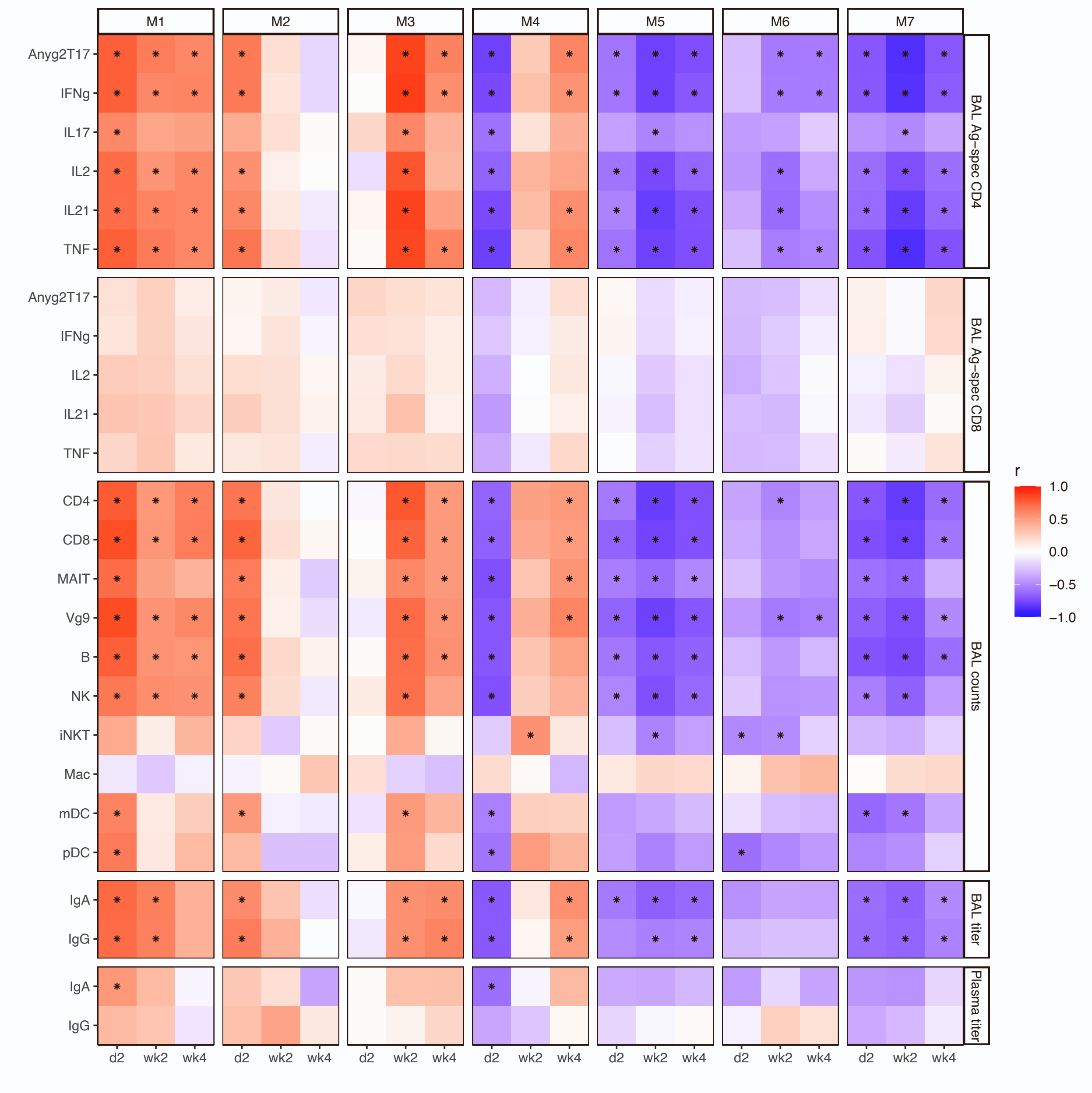

Figure S3. Complete correlation matrix for module scores in blood and immune responses in BAL or plasma. Pairwise pearson correlation coefficients (r) are shown between summary scores for each module at each timepoint and various immune responses of interest. Correlation p-values were adjusted using the Benjamini-Hochberg method; significant correlations at a false discovery rate threshold of 0.01 are indicated with an asterisk. BAL, bronchoalveolar lavage; Ag-spec, antigen-specific; Anyg2T17, expressing any combination of IFNγ, IL-2, TNF, IL-17. Antigen-specific CD4 and CD8 T cell responses are defined as the frequency of BAL CD4 or CD8 T cells expressing a given cytokine upon *ex vivo* restimulation with *Mtb* whole cell lysate eight weeks post-vaccination. BAL counts of various cell types and antibody titers are on a log10 scale and were measured four weeks post-vaccination, with the exception of NK cell counts which were measured eight weeks post-vaccination. pDC cell count measurements were missing for nine (26%) animals. All module scores were normalized to each animal's baseline. Related to Figure 2.

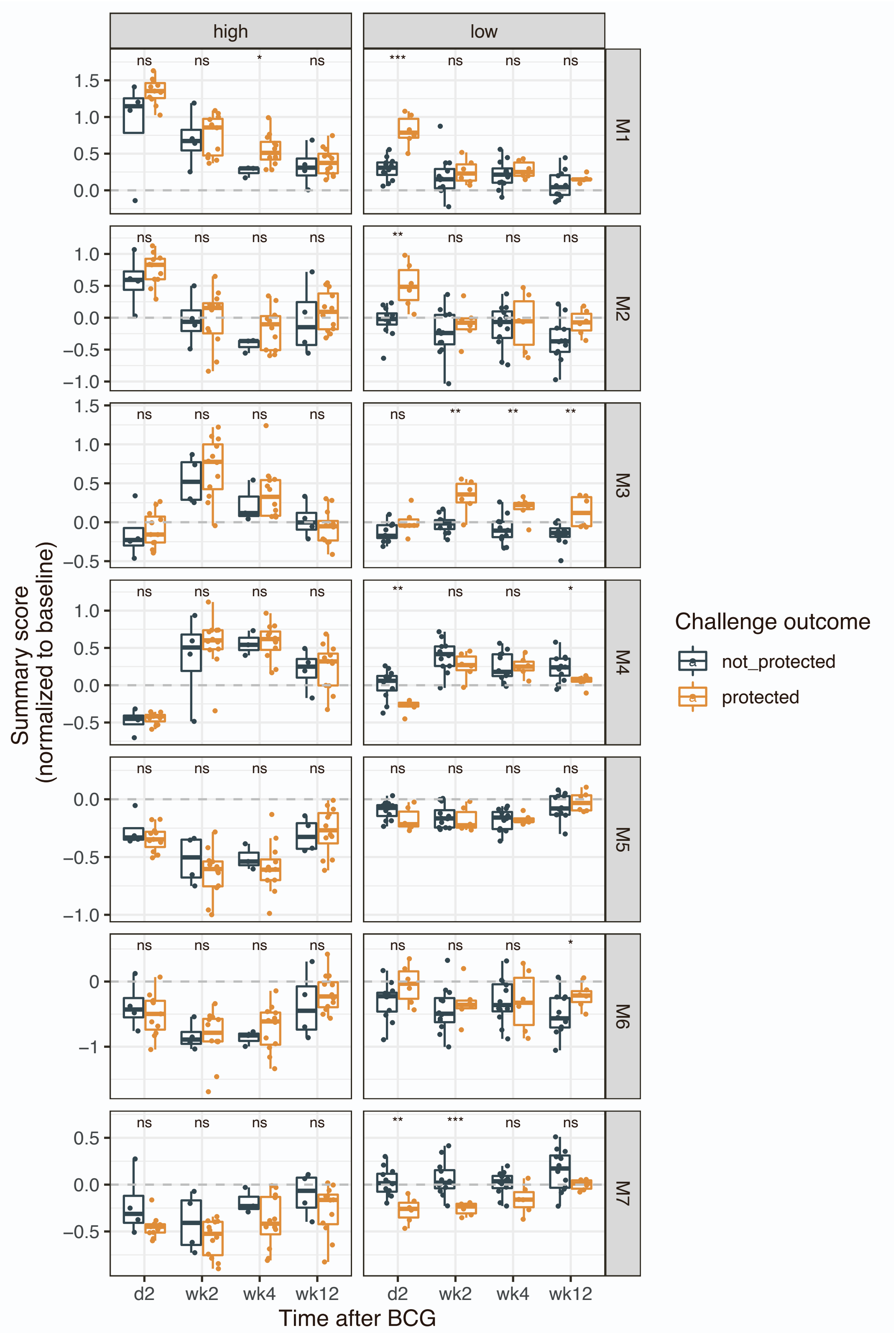

Figure S4. Module activity stratified by IV BCG dose and outcome following challenge. Summary scores (normalized to baseline) of all seven modules over time following vaccination, stratified by IV BCG dose and whether macaques were protected or not protected following *Mtb* challenge. The dotted horizontal line at y=0 indicates no change from baseline. Wilcoxon p-values are shown: *p ≤ 0.05, **p ≤ 0.01, ***p ≤ 0.001. Related to Figure 3b.

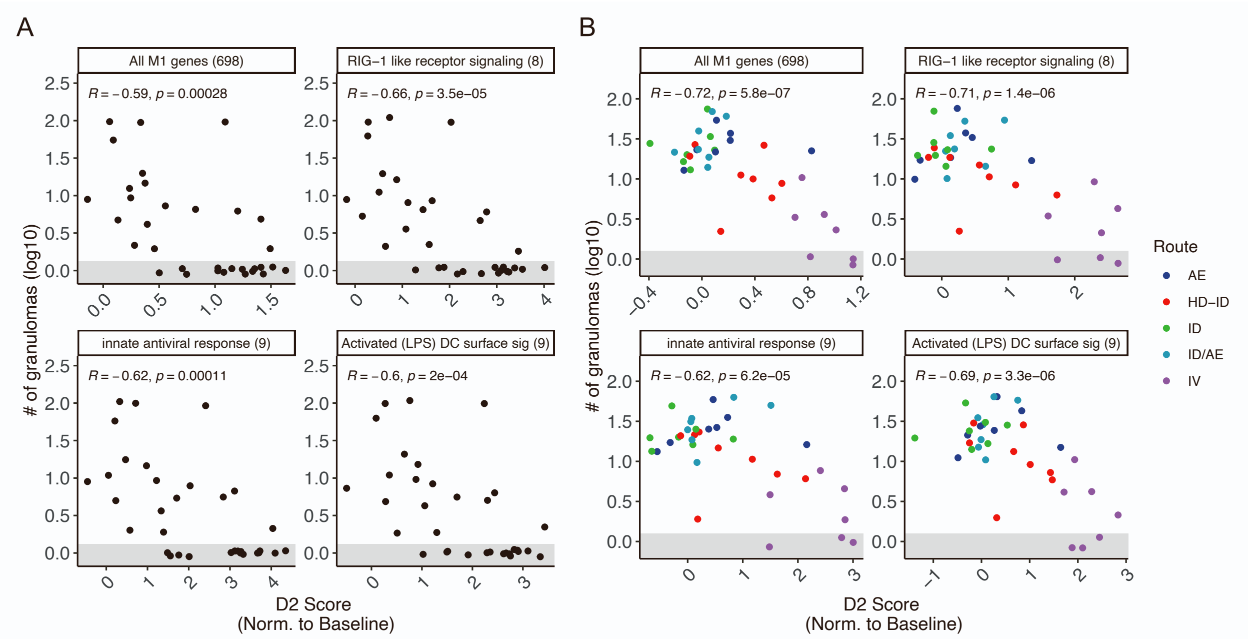

Figure S5. Correlations between the granuloma burden following challenge and module 1 pathways at day 2 post-vaccination. Correlations between the number of granulomas (log10) upon necropsy and day 2 scores of module 1 or its sub-pathways in the A) dose study cohort or B) route study cohort. Pearson correlation coefficients and corresponding p-values are shown. The number of genes in each set is shown in parentheses to the right of the set name. All scores were normalized to each animal's baseline. Data points are jittered to reduce overplotting; all points in the shaded grey area represent animals with no detectable CFUs. Related to Figure 3e-f.
